# Strange Little Flies in the Big City: Exotic Flower-Breeding Drosophilidae (Diptera) in Urban Los Angeles

**DOI:** 10.1371/journal.pone.0122575

**Published:** 2015-04-29

**Authors:** David Grimaldi, Paul S. Ginsberg, Lesley Thayer, Shane McEvey, Martin Hauser, Michael Turelli, Brian Brown

**Affiliations:** 1 Division of Invertebrate Zoology, American Museum of Natural History, New York, New York, 10024–5192, United States of America; 2 Department of Evolution and Ecology, University of California Davis, Davis, California, United States of America; 3 Department of Entomology, The Australian Museum, Sydney, New South Wales, Australia; 4 California Department of Food and Agriculture, Sacramento, California, United States of America; 5 Entomology Section, Natural History Museum of Los Angeles County, Los Angeles, California, United States of America; University of Arkansas, UNITED STATES

## Abstract

Urban landscapes are commonly considered too mundane and corrupted to be biotically interesting. Recent insect surveys employing 29 Malaise traps throughout Los Angeles, California, however, have uncovered breeding populations of two unexpected species of one of the most studied and familiar groups of organisms, *Drosophila* “fruit” flies. Unlike most introduced species of drosophilids, which breed in fresh or decaying fruits, these are specialized flower-breeders. A common species in the survey was *Drosophila (Drosophila) gentica* Wheeler and Takada, previously collected only once, in El Salvador. It belongs to the *flavopilosa* species group, all species of which have been known until now from central Chile, Argentina and Uruguay, to Veracruz, Mexico and the Caribbean, breeding in flowers of *Cestrum* (“jessamine”) and *Sessea* (Solanaceae). The Los Angeles populations are probably breeding in a native and/or introduced *Cestrum*; in addition, populations in San Luis Obispo County were visiting ornamental *Cestrum*. *Drosophila gentica* occurs as far north as San Francisco, where it was found breeding in *Cestrum aurantiacum*. *D*. *gentica* is redescribed and figured in detail for diagnostic and identification purposes. Specimens from Jamaica previously identified as *D*. *gentica* are a distinct species but are not formally described in lieu of complete male specimens. Rare in the Malaise traps was *Drosophila (Sophophora) flavohirta* Malloch, a common species in Australia on the blossoms of native Myrtaceae, found on introduced *Eucalyptus* in South Africa and both *Eucalyptus* and *Syzygium* in Madagascar; adults feed on myrtaceous pollen and nectar, larvae breed in the flowers. It is also redescribed in detail, including its unusual egg. This is the first New World report of this species; DNA sequences confirm it is a morphologically highly aberrant member of the *D*. *melanogaster* species group. This study reveals how intensive field sampling can uncover remarkable biodiversity in even the most urbanized areas.

## Introduction

As urban green areas become better recognized for improving quality of life, with parks and lots replanted with native species, and urban/community gardens multiplying, more attention is being paid to species within cities [1]. Given their great diversity and ecological significance, as pollinators in particular, arthropods are a major focus besides the plants, or at least should be [2]. For example, in a 4-year survey of the community gardens in the Bronx and East Harlem in New York City, 54 species of bees were found, some 13% of the entire New York State fauna [3]. Among these 54 bee species, 19% were exotics.

Despite the loss of most native species [4], urban areas can surprisingly yield even new species. In 2002, for example, a new genus of tiny millipede was found in New York’s Central Park. The millipede was never officially described, although a name (*Nannarup hoffmani*—a *nomen nudum*) appeared in the *New York Times* article that announced the discovery [5]. As expected, many new urban species are actually introductions; “Nannarup”, for instance, putatively belonging to an Asian lineage, was probably introduced with soil from plantings. Newly discovered urbanites, however, can be indigenous, and familiar urbanites can be cryptic new species. Perhaps the most remarkable example of the latter is the recent report of a new morphocryptic species of leopard frog, *Rana kauffeldi* Feinberg et al., from northern New Jersey, southeastern (Hudson) New York, and one of the boroughs of New York City, Staten Island [6, 7]. Identification of this unique frog was based on nuclear and mitochondrial DNA sequences, mating calls, and some morphological features.

Here, we present two new, equally remarkable examples of hidden and unexpected biodiversity, but which involve drosophilid flies, thriving amidst one of the most populated urban centers in the world, Los Angeles, California. *Drosophila* is such an intensively studied eukaryote that it is perhaps the sole insect whose genus name is familiar to much of the general public. In an area housing over 1,000 people/km^2^, it is astonishing that a survey would uncover two unusual drosophilid species, one previously collected only once (in Central America), the other known only from the Southern Hemisphere. Neither of these drosophilids was found by a *Drosophila* survey in Los Angeles four years earlier [8], in which another *Drosophila* new to California was documented, *D*. *bifurca* Patterson and Wheeler (albeit a species previously known from the southwest U.S. and northern Mexico) [9]. Notably, our new survey used Malaise traps, which rely on passive interception of flying insects. In contrast, other surveys (e.g., [8–10]) relied on banana-yeast baited traps, traditionally used to capture frugivorous drosophilids.

The common names for Drosophilidae, “fruit flies” or “pomace flies”, belie the true ecological diversity of the family. In fact, the range of larval hosts of drosophilids is extraordinary [11], and one of the more common lifestyles involves breeding in flowers [12]. An exemplar of flower-breeding drosophilids has been the *Drosophila flavopilosa* species group, one topic of our report, a lineage of 17 described species and at least 10 undescribed ones that occur throughout the neotropics from Veracruz, Mexico to central Chile, and the Caribbean islands [13–24]. The other discovery reported here is a highly unusual member of the *Drosophila melanogaster* species group. While originally thought to be found only in *Eucalyptus* flowers and therefore native to Australia and introduced into South Africa and Madagascar [25, 26], the presence of *D*. *flavohirta* in other myrtaceous genera native outside of Australia opens the question of the geographical origin of this species. As predicted [26], *Drosophila flavohirta* Malloch appears to be spreading to parts of the world where eucalypts have been introduced and cultivated; this report is the first known New World occurrence.

Our report of these two species has a primarily ecological and systematic emphasis. While it is highly unlikely that *Drosophila gentica* can be a pest on tomato, potato, peppers or other solanaceous crops, reporting their North American occurrence is important since some drosophilid introductions have had unexpected consequences. The most dramatic such consequence concerns the spotted-winged fruit fly, *Drosophila suzukii* (Matsumura). For many years this fly was a well-known major pest of cultivated fruit crops in its native Japan [27]. It appears to have been introduced into California in approximately 2008 and within three years spread throughout the southern half of North America up to Ontario [28], as well as into southern Europe [29]. It is a serious agricultural pest in its introduced range, particularly of small, soft berries and drupes [30], and it is even common in hardwood forests of the eastern U.S. (D. Grimaldi, pers. observ.). It is now also established in southern Brazil [31]. Given the temperate climate of Japan, establishment of *D*. *suzukii* in North America and Europe is not surprising, although the speed of its spread was remarkable.

Another important drosophilid invasion was by *Zaprionus indianus* Gupta, native to Africa, the Middle East, and India, where it is a highly polyphagous breeder in fermenting fruits. It was first detected in the New World in São Paulo, Brazil in 1999 [32] and had spread to Panama by 2003 and Florida by 2005 [33]. Now it is a significant agricultural pest in Brazil, particularly on figs (*Ficus* sp.), and it even appears to be adversely affecting populations there of native and other introduced frugivorous drosophilids [34]. Specimens are occasionally found as far north as New York City, although it is unknown if the species is breeding there since the species seems limited to tropical and subtropical climates.

One of the main features of *Drosophila suzukii* and *Zaprionus indianus*, indeed of all major invasive drosophilids—like *Drosophila melanogaster* Meigen, *simulans* Sturtevant, *busckii* Coquillett, *repleta* Woollaston, *hydei* Sturtevant, and *funebris* (Fabricius)—is their polyphagy and fecundity. Each of them can breed quickly on a wide array of fresh, damaged, or decaying fruits and organic substrates (e.g., [10]). As invasives, *Drosophila gentica* and *D*. *flavohirta* are unique in their host specialization and apparent low fecundity, laying a single large, very mature egg at a time, a common feature of anthophagous Drosophilidae. *Drosophila gentica*, in fact, was the second most abundant species in our Malaise trap samples from Los Angeles, after the common, cosmopolitan invasive *Drosophila simulans*. *Drosophila flavohirta* in South Africa actually appears to be a competitor with *Apis mellifera* Linn., the presence of fly larvae in the flowers making them unattractive to bees and negatively impacting eucalypt honey production [25, 35–37].

## Methods, Materials, Results

**Fig 1:**
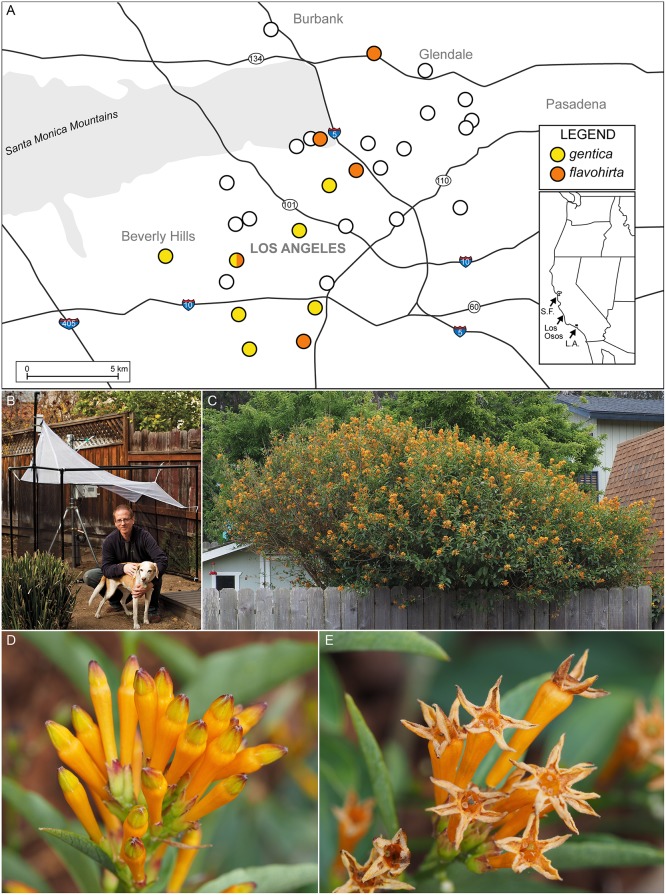
Contexts for sampling flower-breeding *Drosophila* in Los Angeles and other areas in California. **A.** Map of the Los Angeles area, showing major highways, each dot indicating the location of Malaise traps used in the BioSCAN project. Yellow dots are where *Drosophila gentica* specimens were captured; orange dots are where *D*. *flavohirta* specimens were captured; there is one site where both species were captured. Inset shows areas outside of Los Angeles where *D*. *gentica* was also found. Map: **B.** Malaise trap in backyard with two of the participants, one of them Eric Keller. Photo by Phyllis Sun. **C-E**. ***Cestrum nocturnum* x *diurnum* hybrid** (“Orangel Peel”) in flower, May 2014, Los Osos, California. The shrub (C), was attracting hundreds of *Drosophila gentica* to its small tubular flowers (D, E). Photos by Brian Brown.

Specimens of both *Drosophila* species were originally collected as part of the BioSCAN project; *D*. *gentica* was subsequently found also on host plants. BioSCAN is an innovative project based at the Natural History Museum of Los Angeles County designed to survey urban biodiversity using a collecting method, Malaise traps, usually deployed in more natural habitats, while also engaging the general public in the sampling program. At each of 29 sites across the City of Los Angeles, California, a Malaise trap and weather station were set up (Fig 1). Almost all sites are backyards of private citizens who volunteered to host traps for the study; additionally, one is a school, one is the Natural History Museum of Los Angeles County (LACM) “Nature Garden”, and one is a community food garden. Express, written permission for the set up and maintenance of the traps by the landowners is on file at the Natural History Museum of Los Angeles County. No endangered or threatened species were involved. Geographic coordinates are given in the Material Examined section of the species treatments, summarized in the map in Fig 1. Malaise traps are tent-like structures made of a very fine-screened fabric, into which flying insects are intercepted and collected (Fig 1B). The traps can be set up for more than a year and emptied on a regular basis; they often collect insect species rarely captured using hand netting or other traditional methods. The diversity of focal groups of insects in the BioSCAN Malaise traps is being analyzed in their association with various indices of urbanization and land use to assess the effects of the city on the insect fauna. Additionally, besides surveying focal taxa, samples are screened for unusual taxa such as the *Drosophila* reported herein. Our new *Drosophila* records from the Malaise traps were collected and identified in the first three months of sampling of the BioSCAN project, during the winter when insect populations and diversity are lowest. Therefore, further unrecorded species are expected in summer samples.

All measured specimens were point mounted. In contrast to older specimens, new specimens were preserved in 70% ethanol treated with the solvent hexamethyldisilixane (HMDS), which preserves the color of specimens with more fidelity, also making them more fully distended and cleaner. Older museum specimens dried directly after capture or straight from ethanol are slightly greasy and appear slightly darker. Measurements of point-mounted specimens were generally made at 60-100X using a Nikon SMZ 1500 stereoscope with a Nikon DSRi1 digital camera and NIS Elements software; error range is approximately ± 0.01 mm (including the variation due to positioning the specimen). Standard measurements and ratios were made as given in [38]. A total of 43 measurements were made for each of five specimens from LA, five paratypes of *D*. *gentica* from El Salvador, and the four specimens of “*gentica*-like” from Jamaica (in [13]). All six specimens of *D*. *flavohirta* from LA were measured, and compared with measurements of five randomly sampled specimens collected 28 December, 2010 by S. McEvey in Stroud, New South Wales, Australia, off of *Syzygium* flowers (32.4079°S, 151.9672°E). Variation in measurements and ratios between samples of native and introduced specimens are given in the redescriptions only where significant (p<0.05) as based on a pairwise ANOVA for equal or unequal sample sizes. Male and female terminalia were dissected in representative specimens from all localities. Terminalia were macerated in warm 10% KOH, rinsed in water and 70% ethanol, dissected in glycerine using fine tungsten needles, and mounted on microscope slides in glycerine jelly for observation at 100-400X.

One of us (L. T.) collected flies on inflorescences in the Los Angeles area between 11–17 July, 2014, to assess potential hosts of the introduced species. The areas visited were: Mildred E. Mathis Botanical Garden (34°03'54.4"N, 118°26'28.4"W), the University of California, Los Angeles campus (34°04'12.2"N, 118°26'34.3"W), Tongva Park (34°00'41.4"N, 118°29'38.3"W), Palisades Park (34°00'00.8"N, 118°29'08.7"W to 34°01'32.8"N, 118°30'50.5"W), Los Angeles Arboretum and Botanical Garden (34°08'28.6"N, 118°03'10.4"W), Huntington Gardens (34°07'51.0"N, 118°06'52.0"W), and Rivas Canyon Park (34°03'25.0"N, 118°31'01.1"W). Flies were collected from *Cestrum* as well as various taxa of monocots, especially palms (Arecaceae), our thoughts at the time being that the species later identified as *D*. *flavohirta* might be a palm-breeding species. Although *Cestrum* sp. was found in two LA localities, only an anthomyiid and *Thaumatomyia* (Chloropidae) were found on these. Collecting by B.B. on *Cestrum nocturnum* in Los Angeles also found no *D*. *gentica* visitors. A species of Myrtaceae was sampled in LA (*Melaleuca nesophila*, or “showy honey myrtle”, native to Australia), on which only *Milichiella* sp. (Milichiidae) was found but no *D*. *flavohirta*. *Thaumatomyia* sp. was also found on *Trachycarpus* (Arecaceae) and *Polianthes tuberosae* (Asparagaceae); and another chloropid (*Fiebrigella* sp.) on *Brahea nitida* (Arecaceae). Anthomyiidae were collected from *Yucca* (Asparagaceae)(as was a specimen of *Leucopis*: Chamaemyiidae), and *Dietes iridoides* (Iridaceae). Another genus of Milichiidae (*Milichia* sp.) was found on *Brahea brandegeei* (Arecaeae).

One of us (M.T., assisted by W. M. Baca) searched specifically for *D*. *gentica* on *Cestrum* species at the University of California, Davis arboretum, the University of California Botanical Garden, Berkeley, and the San Francisco Botanical Garden (SFBG). On 30 July, 2014, *D*. *gentica* was observed in the SFBG (37°46’1.38”N, 122°28’11.45”W) by W. M. Baca on *Cestrum aurantiacum*. Several adult flies were collected and returned to U.C. Davis along with *C*. *aurantiacum* flowers. Adult *D*. *gentica* emerged from the collected flowers. Hence, the breeding range of *D*. *gentica* within California extends northward to at least San Francisco. Months of systematic sampling on flowers of *Cestrum* and various Myrtaceae throughout California are required to thoroughly assess host use and geographic distributions of the two drosophilid species.

In order to genetically determine the species identity of *D*. *flavohirta* in California, DNA was compared with specimens collected by S.M. in Stroud, Australia (locality data given above). DNA was extracted from a female *flavohirta* from sample #15778, and a female from Stroud, Australia, using standard ethanol extraction from which COI partial coding sequences were obtained and compared. Universal primers 5’-CCTACAGGAATTAAAATTTTTAGA-3’ and 5’- TCCAATGCACTAATCTGCCATAT-3’ were used to amplify a ca. 600-bp region of the COI CDS [39]. PCR conditions were as follows: denaturation at 94°C for 30 sec., annealing at 55°C for 30 sec., and elongation at 72°C for 30 sec for 35 cycles. Sequence alignments were performed using MUSCLE 3.8 [40].

Alignment of the LA and Stroud, Australia sequences showed 98.2% identity. This level of divergence within the amplified region is typical of intraspecific divergence within various species of the *D*. *melanogaster* species group. For example, a standard nucleotide BLAST of the *D*. *simulans* sequence, homologous to that amplified from the *flavohirta* samples, revealed nucleotide identities ranging from 98.0–100% amongst populations of *simulans* (the homologous region was obtained from *D*. *simulans* isolate RU259 complete mitochondrial genome, accession number AF200849.1). Additional collections and analyses are needed to comprehensively determine divergence of *D*. *flavohirta* populations and the placement of *flavohirta* within *Sophophora* [41–43], as discussed below with some new molecular data (S1 Text).

Repositories for the specimens are the Los Angeles County Museum, California Department of Food Agriculture (Sacramento, California), The Australian Museum (Sydney), and American Museum of Natural History (New York).

## Systematics

### The *Drosophila (Drosophila) flavopilosa* Species Group

**Fig 2:**
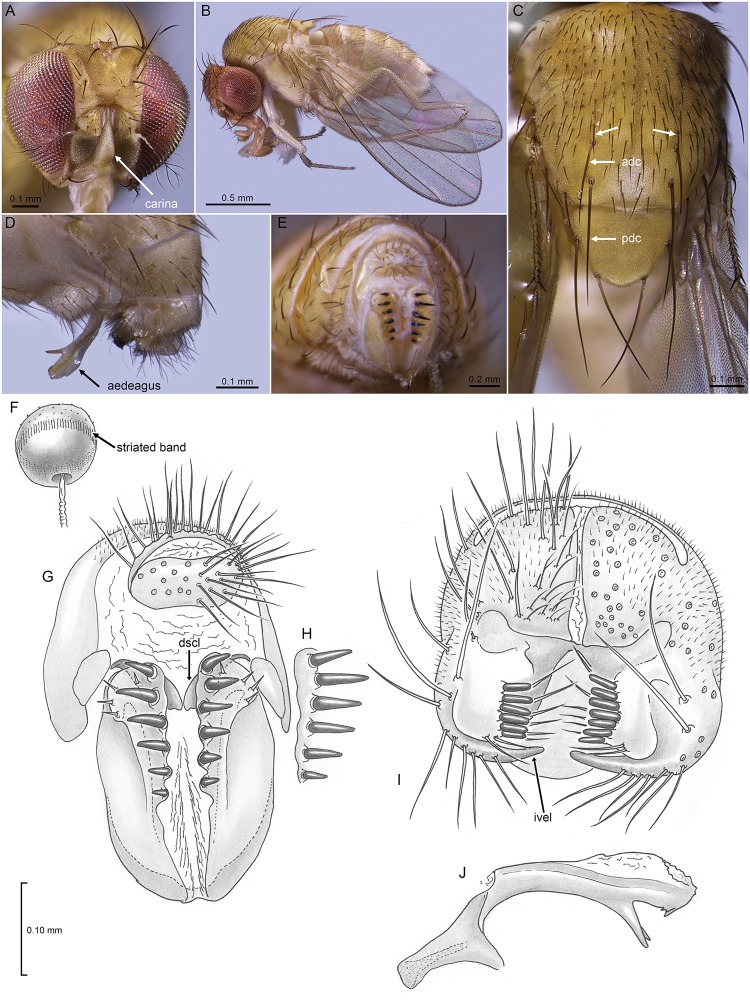
*Drosophila gentica* Wheeler and Takada collected in Los Angeles. **A**: frontal view of head. B: Female, lateral view. **C**: Male, dorsal view of thorax; Unlabelled arrows point to enlarged acrostichals in front of *adc* setae. **D:** Male terminalia, lateral view, with aedeagus everted. **E**: Female terminalia, posterior view. **F:** Spermatheca. **G, H:** Drawings of female terminalia of *Drosophila gentica* specimens from El Salvador (H, right valve) and Los Angeles (G, complete terminalia). I: Male terminalia of LA specimen, posterior view. **J**: Aedeagus of LA specimen (male), lateral view. Abbreviations: *adc*, anterior dorsocentral seta; *dscl*, dorsal sclerite (of oviscapt), *ivel*, inner lobe of ventral epandrial lobe; *pdc*, posterior dorsocentral seta. For more details and comparisons see S1 Text.

The *flavopilosa* group is very distinctive, morphologically and ecologically. All 17 described species are light-bodied flies with faint or no markings (Fig 2; S1 Fig), which contrasts in the female with her remarkable oviscapt of large, heavily sclerotized, black teeth (Fig 2E; S2 Fig). The teeth curve slightly outward, and in some species each tooth is situated on a peduncle; those on opposing valves interdigitate when the valves are pressed together. This structure suggests that the oviscapt may function like a surgical retractor: when the valves separate the teeth grab and separate the flower tissue, allowing an egg to be inserted. Like many species of flower-breeding drosophilids, flies in the *flavopilosa* group lay a single mature egg at one time, rather than a clutch typical of saprophagous species. The eggs either have a pair of very short, stubby subapical filaments, or lack them entirely, again typical of flower-breeders. The larva hatches quickly after oviposition and begins feeding on flower tissue. According to Brncic [12], mature larvae feed on pollen. There is very little information on whether these flies are detrimental to *Cestrum* flowers (e.g., cause premature flower abscission) or may even be beneficial, as pollinators or biocontrol agents of their weedy, toxic plant hosts. Though planted as ornamentals in California, at least in South Africa (and probably other regions) *Cestrum* is considered to be a weedy invasive [44]. Santos and Vilela [24] mentioned that *flavopilosa*-group flies can be “excluded” as pollinators of *Cestrum* since the “adult flies are larger than the diameter of the tiny tubular flowers and therefore unable to enter the corollas.” Observations of *D*. *gentica* by one of us (B.B.) in Los Osos, San Luis Obispo County, CA revealed that the flies readily passed in and out of the corollas of a *Cestrum diurnum* x *nocturnum* hybrid (“Orange Peel”) (Fig 1C–1E).

Molecular phylogenetic studies indicate that the *flavopilosa* group is most closely related to another Neotropical species group of *Drosophila*, the *annulimana* group [45–47], near the base of the “*virilis-repleta*” radiation of *Drosophila*. Species of the *annulimana* group are not light bodied, and they have a typical *Drosophila* oviscapt with a marginal row of small pegs.

***Drosophila (Drosophila) gentica* Wheeler and Takada.** Fig 2; S1–S3 Figs.

*Drosophila gentica* Wheeler and Takada, 1962, *in* Wheeler et al., 1962 [13]: 406.

DIAGNOSIS: Small, thorax length ca. 0.90 (0.82–0.98) mm, body dull yellowish with areas of pale infuscation; basal flagellomere cream, lighter than face, sometimes with ventral half of face darker than rest; no markings on wings; labellum slightly geniculate; apical scutellar setae crossing (Fig 2C). Morphologically distinguished from other species in the *flavopilosa* group most reliably by male terminalia, specifically: aedeagus short, slightly arched in lateral view, apically bulbous, with pair of large preapical spines ventrally, smaller spines and irregular serrations near apex (Fig 2D, 2I and 2J); hypandrium trapezoidal (vs. U-shaped). In addition (♀): bases of oviscapt teeth not tuberculate (Fig 2E, 2G and 2H); spermathecal capsule heavily sclerotized, spherical, without apical “cap,” with equatorial band of short striae (Fig 2F).

DESCRIPTION: See (S1–S3 Figs).

TYPES: Holotype: ♂, “**El Salvador**: San Salvador, Jan. 1954 coll. W. B. Heed”, in U.S. National Museum of Natural History, Washington, D.C. Paratypes: 4♂♂, 4♀♀ [same label data], in American Museum of Natural History, New York. Examined by D.G. All types designated in [13].

NEW MATERIAL: **USA: California, Los Angeles County, Los Angeles**, all collected in Malaise traps and originally preserved in 95% ethanol: **Carthay,** 34.059°N 118.369°W, 29 Oct 2013–5 Nov 2013. BioScan site 19. Coll. Teresa Dahl. [sample] 15325 BioSCAN. 1 ♀; **Koreatown**, 34.072°N 118.291°W, 3 Nov 2013–9 Nov 2013. BioScan site 11. Coll. Peter Ralph [sample] 15339 BioSCAN. 1 ♂ (dissected), 7 ♀♀; **Silverlake**, 34.093°N, 118.274°W, 28 Sep 2013–5 Oct 2013. BioScan site 5. Coll. Walter Renwick. [sample] 15226 BioSCAN. 5 ♂♂ (1 dissected), 2 ♀♀; **University Park**, 34.034°N, 118.281°W, 63 m altitude, 18 Sep 2013–25 Sep 2013. BioScan site 3. Coll. Peggy Hentschke. 15192 BioSCAN, 2 ♂♂ (2 dissected), 3 ♀♀ (1 dissected). In addition, 14 ♀♀ pooled from four samples: **Jefferson Park**, 34.03°N, 118.327°W, 14 Sep 2013–21 Sep 2013. BioScan site 8. Coll. Ray Fujioka. 15185 BioScan, 1 ♀. **Leimert Park**, 34.014°N, 118.321°W, 30 Aug 2013–7 Sep 2013. BioScan site 10. Coll. LaChristian Steptoe. 15216 BioScan, 1 ♀. **University Park**, 34.034°N, 118.281°W, 63 m altitude, 14 Aug 2013–21 Aug 2013. BioScan site 3. Coll. Peggy Hentschke. 15187 BioScan, 6 ♀♀; **University Park**, ibid, 18 Sep 2013–25 Sep 2013. Coll. Peggy Hentschke. 15192 BioScan, 6 ♀♀.

**USA: California, San Luis Obispo County**, Los Osos, 35.32°N, 120.85°W, 9 m. elevation, Coll. B. Brown V/6/2014, in flowers of “Orange Peel” hybrid *Cestrum nocturnum* x *diurnum* (Fig 1C–1E) Eight males, nine females (one each dissected).

COMMENTS: There is some variation between flies from the paratype series from El Salvador and the California collections. Some of the CA flies have a pale brownish band on the oral margin (S1F Fig), although this does not seem to correlate with any other features. Also, the distiphallus of the CA flies in full ventral view is narrow, the tip barely wider than the neck (S3J–S3L Figs); in paratypes the tip is twice the width of the neck. The aedeagal apodeme in CA flies (S3F–S3H Figs) is larger than that in the paratypes, although this feature can have significant intraspecific variation in other drosophilid species. Most specimens from CA have 6 large prensisetae on the surstylus (a few with a fine, sharp 7^th^) (S3A–S3C Fig); paratypes have 7 large prensisetae. Measurements (see description in S1 Text) further indicate that the CA flies have a slightly narrower front (frons), slightly longer posterior dorsocentral seta, and differences in two wing indices. It is unlikely that these differences amount to species differentiation, though molecular comparisons would be very useful when fresh material is available from Central America.

***Drosophila* “*gentica-*like”.** S2D, S2F and S2G Figs.

*Drosophila gentica*-like: Wheeler et al., 1962 [13] (specimens from Jamaica)

SPECIMENS: 2 ♀♀, 2 ♂♂ “Bath, Jamaica, coll. Heed 1956” (as labelled), in AMNH (examined). The specimen labels do not have more specific locality information, but in [13] it is mentioned that specimens were examined from “Hot Mineral Springs near Bath, Jamaica”.

DIAGNOSIS: Very similar to *D*. *gentica* but with the following distinguishing features: Wings slightly shorter, length 1.61 mm (1.53–1.67) (vs. 1.85 [1.66–2.14] in *gentica*), thorax length/wing length 0.53 (vs. 0.49); wing slightly narrower, length/width 2.34 (vs. 2.20); 4-V index 2.13 (vs. 2.05). Facial carina slightly shorter, length/width 2.70 (vs. 3.03 in *gentica*) (S1G Fig). Ratio or1-or3 setae 0.91 (vs. 0.81). As figured by Wheeler et al. ([13]: plate III figs 7–10), aedeagus differs with that of *gentica* by the pair of subapical spines being straight, far apart, divergent and heavily sclerotized (vs. slightly curved, bases touching, parallel, no more sclerotized than rest of aedeagus); microtrichia present on dorsal membrane (vs. absent). Female terminalia very similar to that of the El Salvador *gentica* in spermathecal and oviscapt structure, flies from both localities with 6 long oviscapt teeth lacking pedunculate bases; notable differences are Jamaican specimens with a thin pair of sclerites on dorsal surface of oviscapt membrane (S2D Fig) (vs. broader, plate-like sclerites in *gentica*), oviscapt slightly shorter and broader, and there is a gap between teeth 3–4 (vs. teeth evenly separated in *gentica*) (cf. S2B–S2D Figs).

COMMENTS: In ([13]: p. 407) it was mentioned that there “seem to be differences in the copulatory apparatus [between the El Salvador and Jamaica specimens], but there have been too few males to settle this point.” Diagrammatic as the figures are in [13] (reprinted here, S2F–S2G Figs), the Jamaica specimens indeed appear to be a distinct species, confirmed by the diagnostic female and external differences with mainland *gentica* described above. Unfortunately, the genitalic slide preparations for the only known male specimens from Jamaica are missing, and so this species remains undescribed.

### The *Drosophila (Sophophora) melanogaster* Species Group

Genetic evidence places *D*. *flavohirta* within the *melanogaster* species group, although, as described below, this species is morphologically anomalous for the group and even the subgenus. The *melanogaster* species group is the largest of nine named species groups within *Sophophora*, with more than 180 species [48]. Species groups, including the *melanogaster* group, are typically divided into subgroups. There have been numerous studies on the systematics and relationships of the *melanogaster* group, and three prior molecular phylogenetic studies have included *D*. *flavohirta* [42, 43, 49].

The study by Da Lage et al. [42] was based on a 1485-bp coding sequence of the nuclear *Amyrel* gene (GenBank sequence AY733051; geographic origin not indicated); that of Russo et al. [49] was based on regions of six nuclear genes, including the same *Amyrel* sequence posted in GenBank; some species were not fully sequenced; Barmina and Kopp [43] sequenced 5–14 nuclear and mitochondrial loci. In [42], relationships of *D*. *flavohirta* varied depending on the type of analysis (parsimony and Bayesian). In both types of analyses, as well as in the study by Barmina and Kopp ([43]: see their S1 Text), *D*. *flavohirta* is closely related to the *melanogaster* subgroup, the latter a well-supported, intensively-studied lineage of nine species that includes such familiar species as *D*. *melanogaster* Meigen, *D*. *simulans* Sturtevant, and *D*. *sechellia* Tsacas and Bächli. The consensus parsimony tree in [42] was the following: *elegans* subgroup (*takahashii* s.g. (*flavohirta* (*ficusphila* Kikkawa and Peng + *melanogaster* s.g.))); the Bayesian tree consisted of the following grouping: *elegans* s.g. (*ficusphila* + *takahashii* s.g.) (*flavohirta* + *melanogaster* s.g.). In both hypotheses the support values (Bremer, bootstraps, posterior probability) for relationships of *flavohirta* and *ficusphila* were very low.

In [49], the maximum-likelihood tree consisted of the following: *elegans* s.g. (*ficusphila* (*melanogaster* s.g. (*eugracilis* Bock and Wheeler (*takahashii* s.g. (*flavohirta* + *levii* Tsacas))))). *Drosophila levii* (put in the *takahashii* s.g. by [42]) is from New Caledonia, and was placed on morphological grounds with *ficusphila* into a separate small group [50]; these authors [50] did not study *flavohirta*. We have expanded the molecular data set to more confidently place *D*. *flavohirta* with a Bayesian phylogenetic analysis (see S1 Text).

Overall, molecular phylogenetic evidence consistently places *D*. *flavohirta* within the *melanogaster* group, and two published studies, plus our new molecular analysis (S1 Text), converge on a close relationship of this species to the *melanogaster* subgroup, despite a morphology that is highly divergent with this clade. Another biologically interesting aspect of this grouping is that *D*. *flavohirta* is the only *melanogaster*-group species in Australia that is strictly anthophilous, not at all attracted to fruit baits. The five species in the southeast Asian *elegans* subgroup, also in the *melanogaster* species group, breed in various large, tubular flowers, like *Ipomoea* (Convolvulaceae) and *Brugmansia* (*Solanaceae*) [51]; interestingly, at least some of these species can be bred on standard *Drosophila* cornmeal lab medium, whereas most anthophagous species cannot. *Drosophila ficusphila*, another putative close relative of *D*. *flavohirta* from some analyses, is attracted to fruit baits (as the name reflects, it has a predilection for figs); all of these species are typical *melanogaster*-group species that possess male protarsomere sex combs and an oviscapt with sclerotized pegs.

**Fig 3:**
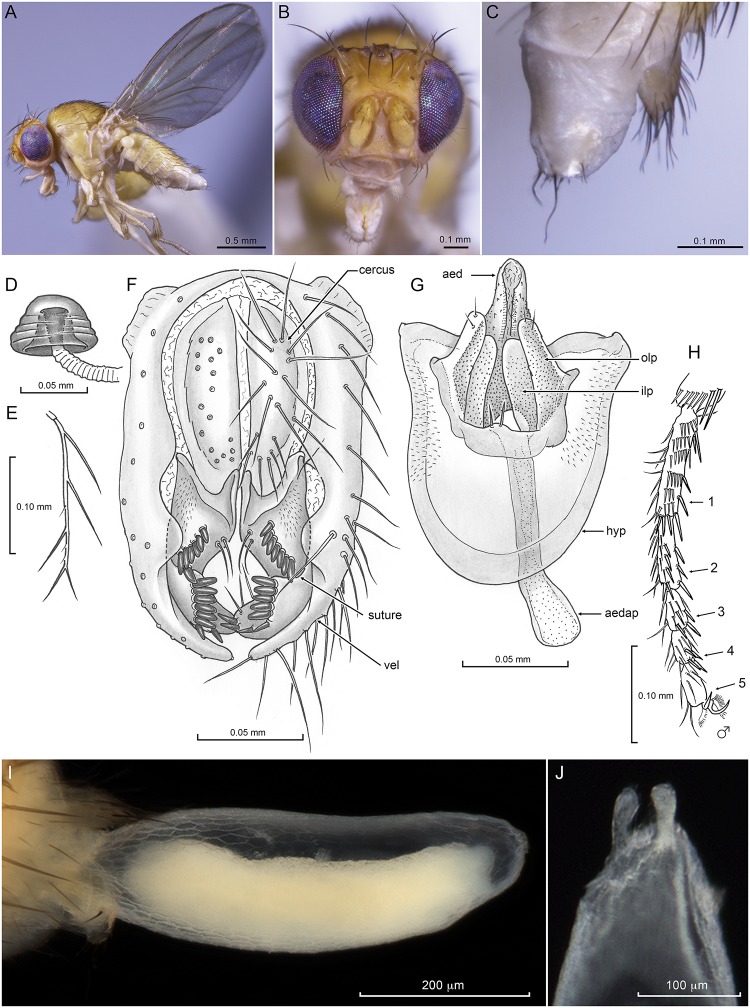
*Drosophila (Sophophora) flavohirta* Malloch. **S**pecimens dried from ethanol using HMDS; **A-H** from California (Photos and drawings by D. Grimaldi), **I-J** from Stroud, Australia (Photos by S. McEvey). **A**. Lateral habitus, female. **B.** Frontal view of head, same. **C**. Female terminalia, lateral view. **D:** Spermathecal capsule, lateral view. **E:** Arista. **F**: Epandrium with cerci and surstyli, posterior view. **G**. Hypandrium, aedeagus, paraphyses and aedeagal apodeme, ventral view. **H**. Male protarsus, mesal view. **I**. Egg emerging from oviscapt. **J**. Anterior tip of egg, showing short, blunt pair of filaments. Abbreviations: *aed*, aedeagus; *aedap*, aedeagal apodeme; *hyp*, hypandrium; *ilp*, inner lobe of paraphysis; *olp*, outer lobe of paraphysis; *vel*, ventral epandrial lobe.

***Drosophila (Sophophora) flavohirta* Malloch.** Fig 3, S4–S7 Figs.

*Drosophila flavohirta* Malloch, 1924: 354. Type locality: Como, New South Wales, Australia. Holotype female in Australian Museum, Sydney (AM K73328).

DIAGNOSIS: Body light yellow with very pale setae; eyes iridescent [color varying with preservation, see below], micropubescent; face slightly raised but not carinate; cheek relatively deep; arista with short braches, 1 ventral and 2–3 dorsal branches, no micropubescence; anterior reclinate orbital seta lateral to proclinate, relatively large, 0.7X length of proclinate; two pairs of dorsocentral setae, no prescutellars; male without thick, black sex-comb setae on protarsus; oviscapt developed, but with only fine apical setae (no pegs); spermathecal capsule short, heavily sclerotized, with well-developed introvert. Male terminalia with ventral lobe of epandrium and surstylus long and pendulous; epandrium without microtrichia, not connected to cercus; surstylus 2-segmented, with 2 rows of prensisetae, rows separated by suture; hypandrium with microtrichia; paraphysis digitiform, bilobed, with short row of fine setulae on outer lobe; aedeagus short, conical, finely textured; aedeagal apodeme long, rod-like; ejaculatory apodeme apparently absent.

DESCRIPTION: See S1 Text.

MATERIAL: **USA, California, Los Angeles County**, **Los Angeles, Silverlake**: Malaise trap site 19 (34.05925°N, 118.3688°W), 28 Dec. 2013–4 Jan. 2014, [sample no.] 15438 # sex; Malaise trap site 19 [ibid.], 28 Jan. 2014–4 Feb. 2014, [sample no.] 15593, 1 female (dissected, in LACM), both specimens collected by Teresa Dahl; **Hollywood**: Malaise trap site 16 (34.09531°N, 118.33351°W) 4 Jan. 2014–18 Jan. 2014, [sample no.] 15608, 1 female (in LACM), collected by Tony Hein. Malaise trap site 6 (34.116°N, 118.2794°W), 1 male (dissected, in LACM), collected by Jeff and Adilia Koch. **Silverlake**: Malaise trap site 7 (34.102°N, 118.257°W) 14.-21.IX.2013, coll. Joe Hogg [sample no.] 15178 (in LACM) 1 male.

**AUSTRALIA: New South Wales**: Stroud, Stroud Garden (32.4079°S, 151.9672°E)., 28 December, 2010, on flowers of *Syzygium* (Myrtaceae), collected by S. F. McEvey. Five males, five females (2 of each dissected). Also, same locality, except collected on flowers of *Callistemon pallidus* (Myrtaceae), by S. F. McEvey. **New South Wales**: Como, near Sydney, on flowers of *Angophora costata* (Myrtaceae), 5 mles, 5 females, collected by S. F. McEvey.

COMMENTS: A short redescription of the species [41] was based on specimens from New South Wales, Australian Capital Territory, Queensland, and Northern Territory, Australia, most details of which are consistent with our redescription. Bock [41] did not mention the unique female terminalia, but described and figured the male genitalia, even commenting [41] that “On structure of male genitalia this species is clearly closely allied to the *melanogaster* species-group. It differs from other species of the group in its highly unusual coloration and in the absence of a sex-comb in the male” (pg. 19). Indeed, the protarsomere setae in males and females are essentially identical and only slightly thicker than remaining setae on the legs (Fig 3H; S6D and S6E Figs); this species lacks the comb of thickened, blunt, sclerotized setae distinctive to almost all other *melanogaster* group species *contra* [43] (their Table S1). There is a host of additional features in *flavohirta* that differ from the *melanogaster* group, in fact probably all species in *Sophophora*. These include the following (S6–S8 Figs):

- Arista with a few, short branches (2–3 dorsal, 1 ventral); vs. generally >3 dorsal and 2 ventral ones.
- Face raised, broad, and flat, with antennae lying in shallow scrobe-like structures that are slightly recessed; vs. face with a narrow, short carina.
- Face with one large pair of vibrissal setae; vs. generally (but not always) two pairs of equal or nearly equal size in *Sophophora*;
- Oviscapt uniquely devoid of sclerotized pegs, each valve only with row of 6 fine, short, marginal setae and one very long, subapical seta; vs. sclerotized pegs of varying sizes surrounding most of oviscapt margin.
- Surstylus (male genitalia) pendulous and two-segmented, with prensisetae separated into two rows; vs. surstylus a one-segmented lobe with a single row of prensisetae.
- Ejaculatory apodeme apparently absent; vs. present. This is a significant and potentially important character.
- Egg highly distinctive in *flavohirta* (Fig 3I and 3J; S7 Fig), indeed so far as known unique in *Sophophora* for the subapical pair of filaments reduced to short, stubby lobes; vs. subapical filaments varying from long and filamentous to flattened and paddle-shaped.

Our comparisons of the male and female genitalia of specimens from Los Angeles and from Stroud, NSW, Australia revealed them to be identical (S6–S8 Figs). The presence of this species on *Callistemon* and *Angophora* in Australia (McEvey, pers. observation, 2014) are new generic records of flower visitation, although *Angophora* was once included within the genus *Eucalyptus*. The illustrations of male terminalia by Bock ([41]: Figs 7, 8) differ from our observations of Los Angeles and Stroud, Australia specimens (e.g., Fig 3) by the following features:

- Hypandrium much broader and shorter, L/W = 0.6, with aedeagal apodeme extending well beyond anterior margin of hypandrium (0.55 the apodeme length); vs. hypandrial L/W = 1.05 in our specimens, with only 0.34X the aedeagal apodeme length exposed.
- Paraphysis lobe is quite short, longest portion ca. 0.25X length of aedeagus; vs. 0.70X aedeagal length in our specimens.
- Paraphysis bilobed, but with inner lobe longer; vs. outer lobe longer in our specimens.
- Surstylus one-segmented, short and straight; vs. two-segmented, with suture between the two rows of prensisetae; pendulous and inwardly curved in our specimens.
- Surstylus with ventral row having 9 long prensisetae; vs. 5 short ones in our specimens.
- Epandrial lobe shorter and broader; vs. longer and slender.

In lieu of dissecting the holotype of *flavohirta*, we conclude that the differences in genitalic figures between material we examined and what Bock [41] presented are probably due to the very diagrammatic nature of Bock’s renderings, not differences among species. Despite this, his placement of the species into the *melanogaster* group was accurate.

There are striking similarities between *D*. *flavohirta* and the rarely collected Australasian genus *Baeodrosophila* Wheeler and Takada, and it is likely that some of these features might be convergence based on anthophilic habits (*Baeodrosophila* visit and probably breed in the inflorescences of *Pandanus* “screw pines” [Pandanaceae: Monocotyledonae]). The similarities (and differences) are the few, short aristal branches (though lacking micropubescence in *flavohirta*) that is found on the main branch of the arista in *Baeodrosophila*; anterior reclinate seta lateral to the proclinate (though the former seta is relatively large in *flavohirta*); postpronotum with two large setae; a distinctive aedeagus with a conical shape and granular surface; digitiform paraphysis (though bilobed in *flavohirta*), simple hypandrium with a pair of paramedian setae on the posterior margin (though these are very short and fine in *flavohirta*, quite long in *Baeodrosophila*); the aedeagal apodeme long and rod-like, and—significantly—the absence of an ejaculatory apodeme. *Drosophila flavohirta* differs from *Baeodrosophila* by being slightly larger, without brownish or infuscate integument; having eyes that are micropubescent and with greenish-purple iridescence; face raised and flat instead of carinate; the oviscapt lacking pegs and not apically slender; the spermathecal capsule heavily sclerotized and with an introvert; and various features of the male terminalia: ventral lobe of epandrium long, pendulous; surstylus pendulous, 2-segmented.

## Discussion: Hosts and Invasiveness

Flower-breeding Drosophilidae are very rarely collected in natural areas by sweeping, in Malaise traps, or by other general methods used for collecting insects; they are virtually never captured using fruit baits (D. Grimaldi, S. McEvey, pers. observations). For example, among the thousands of drosophilids amassed during the Zurqui All Diptera Biodiversity Inventory project in Costa Rica (B. Brown, Principal Investigator), collected using all general and many specialized field methods (including Malaise traps), there are only eight *flavopilosa*-group specimens. Flies in the *flavopilosa* group readily avoid detection even though they occur in abundance at flowers of *Cestrum* spp. during appropriate seasons. That substantial numbers of *Drosophila gentica* have been captured in Malaise traps from various spots in Los Angeles, and the species has been found in Los Osos and San Francisco, indicates that the populations are significant in size and well established. Several females from the Malaise samples contained mature eggs, confirming that these are breeding populations, which can be quite large on very local scales. Brncic [12], for example, calculated the density of adult *Drosophila flavopilosa* Frey in an area of *Cestrum parqui* scrub in central Chile, based on larval infestation rates of hundreds of flowers. Allowing for 90% pre-adult mortality, Brncic estimated the density of adults exceeded 30,000 flies per 100 m^2^.

So far as known, all authoritative host records of the *flavopilosa* group indicate that species are restricted to flowers of *Cestrum* (Solanaceae) (references above), a Neotropical and warm temperate genus of some 175 species of trees and shrubs [52]. Santos and Vilela [24] reared two *flavopilosa*-group species from *Sessea brasiliensis* in São Paulo state, southern Brazil, the only host record from this genus of 18 species. Brncic [12] mentioned (pg. 361) that “in Central and tropical America, it seems that other members of the *flavopilosa* group may utilize other flowers,” for which he cited [53]. Unfortunately, several of the drosophilid identifications in [53] are known to be incorrect (e.g., the common, cosmopolitan species *Drosophila immigrans* Sturtevant is described as *D*. *flexipilosa* Pipkin, 1964); thus, identifications in that paper are suspect. Indeed, there are no *flavopilosa* group flies in Pipkin’s material in the U.S. National Museum of Natural History (D. Grimaldi pers. observ.). In a large survey of flower-breeding Drosophilidae from southern and eastern Brazil [54], flowers were collected from 125 plant species in 47 families. No *Cestrum* or *Sessea* were collected, though flowers of other Solanaceae were, including *Brugmansia*, *Brunfelsia*, *Datura*, *Petunia*, *Solanum*, and *Streptosolen*. In that study there were found 28 species of Drosophilidae breeding in 56 of the flower species and 18 of the families, but no specimens of the *flavopilosa* group. It is quite likely that these flies are indeed restricted to breeding in *Cestrum* and *Sessea*. The studies conducted and reviewed by [12] and by [24] indicate that some species of these flies breed in multiple species of *Cestrum*, others appear to be strictly monophagous. As noted, we found large numbers of *D*. *gentica* in the flowers of a *Cestrum* hybrid in Los Osos, CA (breeding them from the flowers was not attempted), and this fly was actually breeding in *Cestrum aurantiacum* in San Francisco. *Cestrum aurantiacum* is introduced into California, its native distribution being Mexico, Costa Rica, Guatemala, and Venezuela. More extensive field collections are needed to clarify the host and geographic range of *D*. *gentica* in California.

Host specialization of *flavopilosa*-group flies on *Cestrum* reflects the chemistry of this plant genus. The flowers, commonly called “sweet jessamine”, are highly fragrant, and the plants are notoriously toxic, particularly the reproductive structures. Ingestion of foliage, fruits, and flowers can be lethal to humans and grazing livestock [55–57]. Among the toxic types of compounds in *Cestrum* are glycosides and alkaloids, the former of which are implicated in acute hypercalcemia, calcinosis, and strynchine-like poisoning in ungulates [58–61]. Brncic [12] mentioned that “filtered homogenates” of *Cestrum* tissues kill the larvae of highly polyphagous, common species like *Drosophila melanogaster*, *simulans*, *immigrans*, *hydei* and *funebris*.

*Cestrum parqui* L’Herr, the most ubiquitous species of the genus in California, was introduced from southern South America into California, and is reported to occur in Los Angeles and Santa Barbara counties, as well as several counties around and including San Francisco [62]. In the Jepson Herbarium at Univ. California, Berkeley, there are specimens collected in 1893 from Napa City (Napa Co.), 1933 in Amador Co., and 1961 in Santa Barbara Co. Univ. California [63]. Interestingly, there is no mention of *C*. *parqui* in the California state flora [64], nor in the first edition of the Jepson Manual, although the second edition [65] indicates it is a “waif/garden escape” species. In Los Angeles *Cestrum parqui* is not widely planted but is a weedy species that readily disperses (P. Rundel, UCLA Bot. Garden, pers. comm. to LT May 2014). The genus *Cestrum* is not mentioned at all in [66]. *Cestrum parqui* has also been introduced toTexas [67].

Purportedly, *C*. *parqui* flowers in these North American locales from July through December. The fly specimens in this study were recovered from traps within this time frame, specifically 14 August to 9 November, 2013. Interestingly, several *C*. *parqui* plants were in flower at the University of California, Davis Arboretum in July and August, 2014, but *Drosophila gentica* was not found on the plants. In Chile, *Cestrum parqui* supports dense populations of *Drosophila flavopilosa*, a fly that occurs throughout southern South America [12, 13]. In addition, there are two native species of *Cestrum* in North America: *Cestrum nocturnum* L., native to the southern U.S. (California, Texas, Louisiana, Georgia, Florida), and which is planted as an ornamental in California, and *Cestrum diurnum* L. in Texas, Florida, and Puerto Rico [67]. The pharmacology of these species has been well investigated.

Our discovery of *D*. *gentica* in California indicates that the ecological niche modeling by [47] may need to be re-assessed. Using four *flavopilosa*-group species that had the best geographic sampling, and 19 environmental variables (analyzed using MAXENT), they determined that the fly distributions appear to be more limited by abiotic factors like temperature and rainfall than by the distribution of various *Cestrum* species. Unfortunately, sampling of *flavopilosa*-group flies is poor, both for hosts as well as geographically. Indeed, there are no published records of these flies for the entire Brazilian Amazon basin nor for the Orinoco Basin, including all of Venezuela and the Guianas, where *Cestrum* is known to also be diverse.

*Drosophila flavohirta* appears to be restricted to flowers of the large family Myrtaceae, which comprises more than 130 genera that are widely distributed in tropical and warm-temperate regions. To date, flies have been collected in large numbers only on flowers of *Eucalyptus* [25, 26, 36, 41] and *Syzygium* [26], and most recently found on *Callistemon pallidus* in Australia, all genera with massed, nectariferous flowers. The genus *Eucalyptus* contains c. 700 species, all native to Australia and nearby islands, various species of which have been introduced around the world, principally as a source of timber or shade trees since some species grow extremely tall and the resin (or “gum”, source of their popular name “gum trees”) makes them more resistant to insect damage [68]. *Syzygium* is a much larger and more widely distributed genus (c. 1200 species, with a center of diversity in southeast Asia); it too has been naturalized around the world, but not as extensively as *Eucalyptus*. Natural distribution of *Callistemon* s.s. includes the entire eastern margin of Australia and portions of southern Australia; this genus is closely related to *Melaleuca* [69].

*Eucalyptus* was introduced into California during the Gold Rush era of the 1850’s, and now there are some 250 species cultivated in the state [63, 68], with *Eucalyptus globulus* (blue gum) the most common species. *Syzygium* (as *Eugenia)* also occurs in California, but is much more limited in distribution and species within the state (some 44 species). Unfortunately, the flowers of *Eucalyptus* and *Eugenia* were not sampled during our study, only the flowers of the myrtaceous genus *Melaleuca*. Also, since the BioSCAN project is restricted to the Los Angeles area, we did not examine Malaise samples from other regions of California. Eventually, sampling should be made directly from myrtaceous flowers througout the state, especially the San Francisco area (an epicenter of California eucalypts), to determine how far *D*. *flavohirta* has spread. Given the diversity of Myrtaceae, especially in the Australasian Region, and knowing that *Drosophila flavohirta* is not restricted to *Eucalyptus*, it is possible that there are closely related species as yet uncollected in that region.

Several questions thus arise: When was *D*. *flavohirta* introduced into California? How far has it spread? Was *Drosophila gentica* introduced into California, or is it native? Does it (and perhaps other *flavopilosa*-group species) occur in other areas in the U.S. outside of California where *Cestrum* occurs? Native U.S. occurrence of *D*. *gentica* is highly doubtful, since it is extremely unlikely that such abundant flies escaped the detection of several large, very active *Drosophila* research groups in the region. In 1928 the famed “fly group” of Thomas Hunt Morgan moved to the California Institute of Technology in Pasadena [70], less than 20 km from most of our collection sites. This group included Alfred Sturtevant, author of the first treatment of North American *Drosophila* [71]. The other group was at the University of Texas, Austin, active from the 1940’s to 1970’s and where Marshall Wheeler was the systematist [13]. *Drosophila flavohirta* is much rarer in Malaise traps than *D*. *gentica*, somewhat expected since it congregates around arboreal blossoms and is rarely found near the ground. *Drosophila flavohirta* might also be rarely encountered if these are the early days of its establishment in California.

## Supporting Information

S1 Fig Photomicrographs of Wheeler and Takada collected in Los Angeles (Fig 1a–1f, 1h–1j), and of *Drosophila* “near *gentica*” from Jamaica (Fig 1g: see text). a, b: Female, lateral views. c, d: male, dorsal views of thorax (c) and abdomen (d). Unlabelled arrows in c point to enlarged acrostichals in front of *adc* setae. e-g: frontal views of head. e, f: Male *D*. *gentica* from LA (note light brown oral band on specimen in f). g: Female specimen from Bath, Jamaica (note broader, shorter carina). h-j: Terminalia of flies from LA. h: Male, lateral view, with aedeagus everted. i: Female, lateral view. j: Female, terminal view. Abbreviations: *adc*, anterior dorsocentral seta; *pdc*, posterior dorsocentral seta. Photos by D. Grimaldi
(TIF)

S2 Fig Terminalia of *Drosophila gentica* and *gentica*-like species. A-E: Female terminalia of *Drosophila gentica* and near-*gentica* from Jamaica. F, G: Original drawings of male genitalia from Wheeler et al. (1962: their figures II-10 and III-9). f: “*gentica*-like” from Jamaica. g: *D*. *gentica* paratype, El Salvador. A-E: Drawings of female terminalia of *Drosophila gentica* specimens from El Salvador (c) and Los Angeles (a, b, e), and “*gentica*-like” species from Jamaica (d). a: Spermatheca of *Drosophila gentica*. b-d: Posterior (terminal) views of oviscapt. b: Terminal abdominal sclerites of *D*. *gentica* from Los Angeles. c: Teeth on left valve of *D*. *gentica* paratype from El Salvador (slightly greater length of teeth is due to a more full-length view). d: *D*. “*gentica*-like” from Jamaica. Note gap between teeth 3 and 4. e: Anterior (dorsal) view of *D*. *gentica* (LA), showing extensive dorsal membrane. Abbreviations: dscl, dorsal sclerite. Most drawings by D. Grimaldi.
(TIF)

S3 Fig Drawings of male terminalia of *Drosophila gentica* specimens from El Salvador (paratype series) (c, d, e, f, i, j) and newly collected from Los Angeles (a, b, g, h, k, l). No male specimens of “*gentica*-like” from Jamaica were available. a: Epandrium and surstyli of LA specimen, posterior view. b, c: Detail of surstyli with *ivel*. b: LA specimen, with *ivel* intact and partially hidden. c: Paratype (El Salvador), with *ivel* disarticulated. d: Subepandrial sclerite of paratype (El Salvador). e: Ejaculatory apodeme in two views, paratype (El Salvador). f-h: Aedeagus plus aedeagal apodeme, lateral view. f: Paratype. g, h: LA specimens. i: Genitalia (aedeagus, hypandrium, aedeagal apodeme) of paratype (El Salvador), ventral view j-l: Apex of aedeagus (distiphallus), ventral view, of paratype (j) and LA specimens (k, l). Abbreviations: *ivel*, inner part of ventral epandrial lobe. Drawings by D. Grimaldi.
(TIF)

S4 Fig Photomicrographs of *Drosophila (Sophophora) flavohirta* Malloch. **S**pecimens dried from ethanol using HMDS; all are from California. A. Lateral habitus. B. Frontal view of head. C. Lateral view of head and anterior portion of thorax. D. Thorax, dorsal view. E. Female terminalia, dorsal view. F. Ibid., lateral view. G. Female terminalia cleared, lateral view, showing spermathecae. H. Epandrium with surstyli. I. Hypandrium, aedeagus, periphallic structures. Photos by D. Grimaldi.
(TIF)

S5 Fig Drawings of terminalia and other characters of *D*. *flavohirta*. Specimens from Los Angeles. A-D: Female terminalia. A. Oviscapt, ventral view. B. Oviscapt, lateral view. C. Spermathecal capsule, lateral view. D. Oviscapt, detail of apex. E, F: Arista of two individuals. F. Arista of female, from sample 15608. G. Arista of male from sample 15438. H. Epandrium with cerci and surstyli, posterior view. I. Ibid., interior view (setae omitted). Abbreviations: *bss*, basal segment of surstylus; *vel*, ventral epandrial lobe. Drawings by D. Grimaldi.
(TIF)

S6 Fig Structures of *Drosophila flavohirta*. A-C: Drawings of internal male genitalia of *D*. *flavohirta* (LA specimens); D-E: male and female protarsi, oblique ventral views (Australian specimens). A. Hypandrium, aedeagus, paraphyses and aedeagal apodeme, ventral view. B. Ibid., dorsal view. C. Ibid., lateral view. D. Male protarsus of *D*. *flavohirta*. E. Female protarsus of *D*. *flavohirta*. Note pairs of slightly larger, but unsclerotized, setae on tarsomeres, and lack of sexual dimorphism in the setation. Abbreviations: *1–5*: protarsomeres 1–5. *aed*, aedeagus; *aedap*, aedeagal apodeme; *hyp*, hypandrium; *ilp*, inner lobe of paraphysis; *olp*, outer lobe of paraphysis; *seps*, subepandrial sclerite. Photos and drawings by D. Grimaldi.
(TIF)

S7 Fig The unusual eggs of *Drosophila flavohirta*. Specimens from Stroud, NSW, Australia. A, B: Female with partially extruded egg. The oviposition of single, large, mature eggs is a typical feature of anthophilous Drosophilidae (note well-developed embryo through the chorion in figs. B, C), as is the reduction of the anterior filaments of the egg. In this species, the pair of egg filaments typical of *Sophophora* are reduced to small, stubby, preapical lobes (Figs. D–I). Photos by S. F. McEvey.
(TIF)

S8 Fig Bayesian phylograms including *Drosophila flavohirta*. The phylograms focus on *melanogaster* group species and various outgroups. They are generated with (A) and without (B) third-position bases. Both analyses place *D*. *flavohirta* as sister to *D*. *eugracilis* with this pair sister to the *melanogaster* subgroup, relative to the other taxa considered. Numbers indicate posterior probabilities for node support. Phylograms by M. Turelli and P. Ginsburg.
(TIF)

S1 Text (DOCX)
