# Supplementary figures and images for: Strange Little Flies in the Big City: Exotic Flower-Breeding Drosophilidae (Diptera) in Urban Los Angeles
Source: PLoS One. 2015 Apr 29;10(4):e0122575. doi: 10.1371/journal.pone.0122575 (PMC4414507; doi:10.1371/journal.pone.0122575)

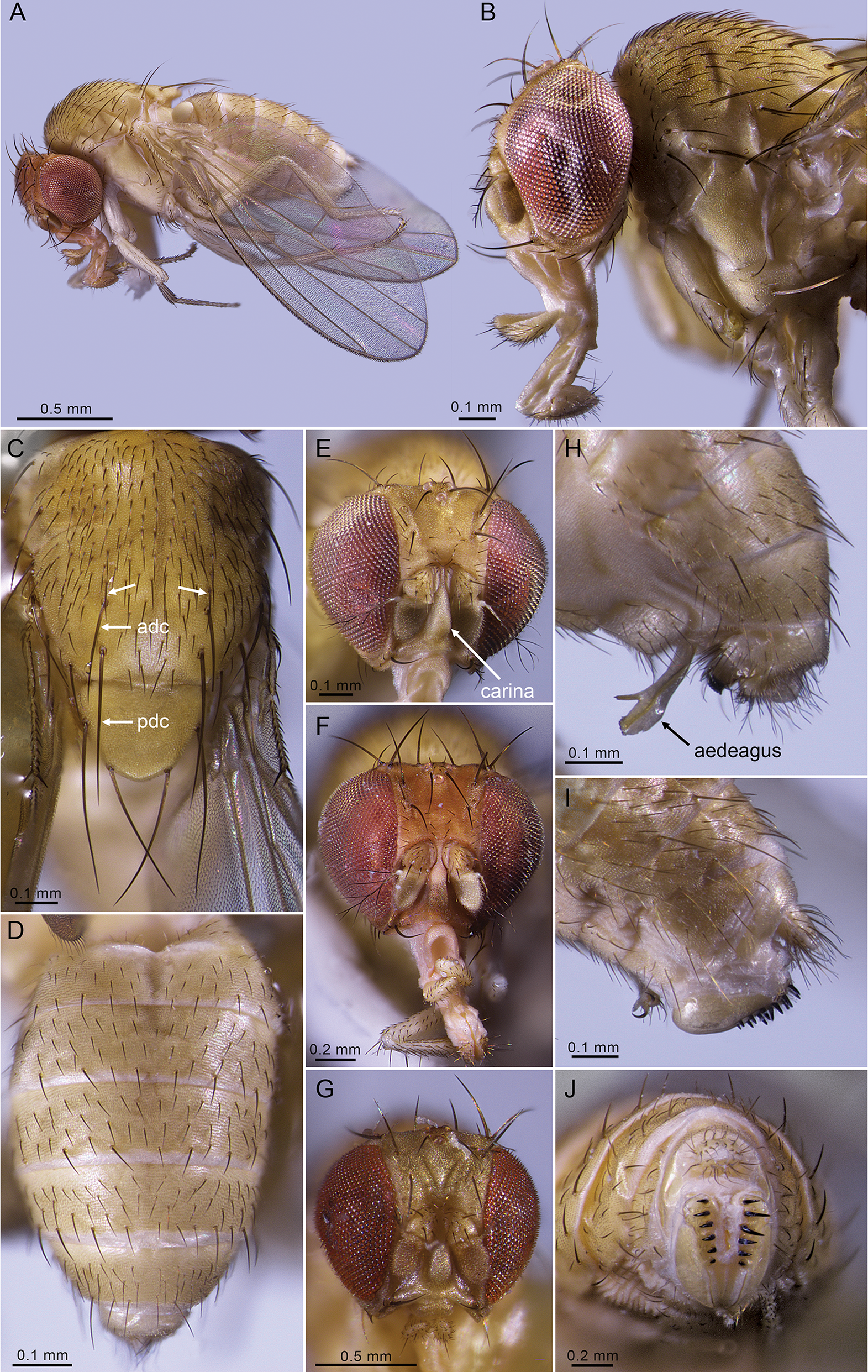

Supplement: S1 Fig — a, b: Female, lateral views. c, d: male, dorsal views of thorax (c) and abdomen (d). Unlabelled arrows in c point to enlarged acrostichals in front of adc setae. e-g: frontal views of head. e, f: Male D. gentica from LA (note light brown oral band on specimen in f). g: Female specimen from Bath, Jamaica (note broader, shorter carina). h-j: Terminalia of flies from LA. h: Male, lateral view, with aedeagus everted. i: Female, lateral view. j: Female, terminal view. Abbreviations: adc, anterior dorsocentral seta; pdc, posterior dorsocentral seta. Photos by D. Grimaldi (TIF) [file pone.0122575.s001.tif]

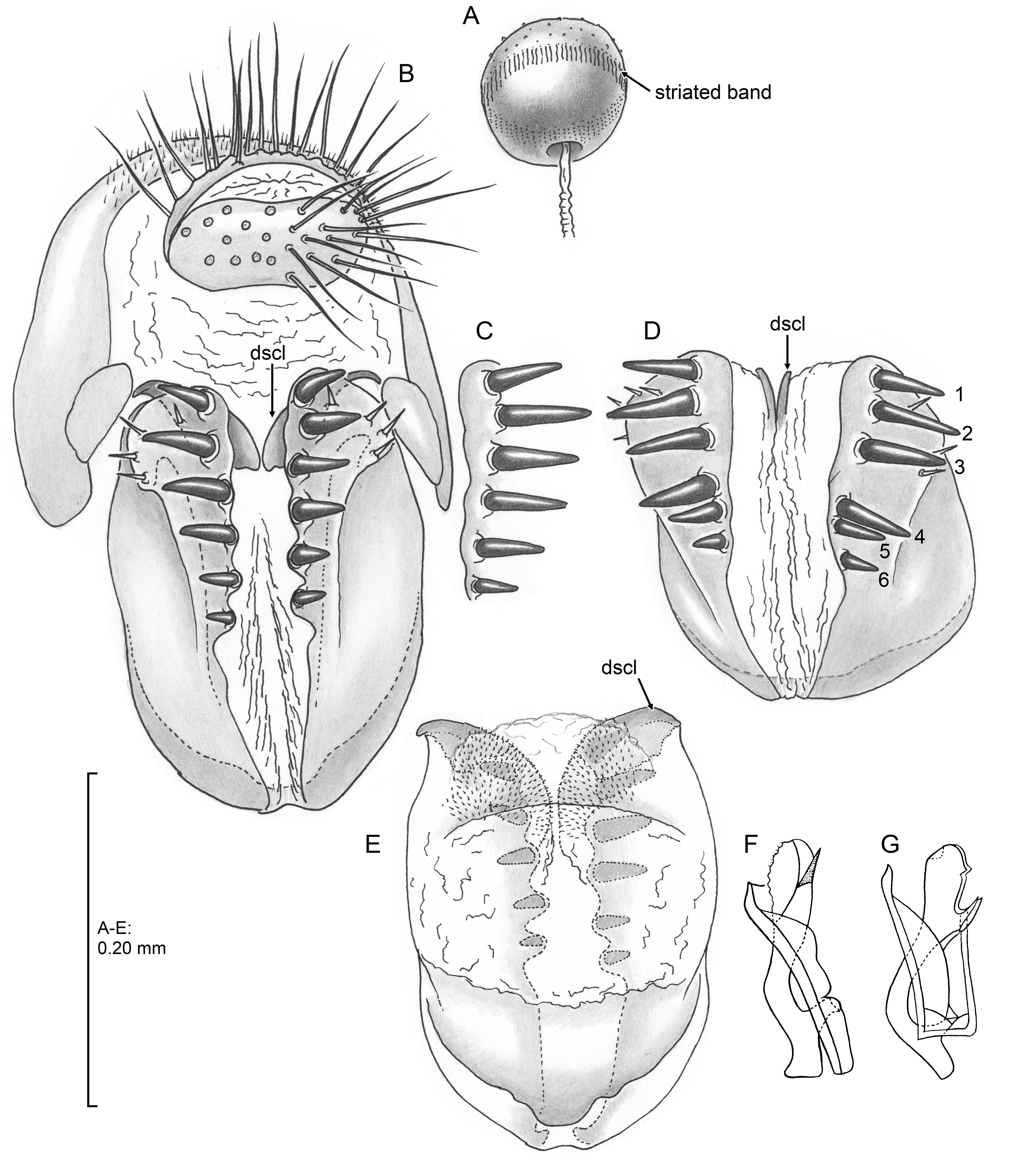

Supplement: S2 Fig — A-E: Female terminalia of Drosophila gentica and near-gentica from Jamaica. F, G: Original drawings of male genitalia from Wheeler et al. (1962: their figures II-10 and III-9). f: “gentica-like” from Jamaica. g: D. gentica paratype, El Salvador. A-E: Drawings of female terminalia of Drosophila gentica specimens from El Salvador (c) and Los Angeles (a, b, e), and “gentica-like” species from Jamaica (d). a: Spermatheca of Drosophila gentica. b-d: Posterior (terminal) views of oviscapt. b: Terminal abdominal sclerites of D. gentica from Los Angeles. c: Teeth on left valve of D. gentica paratype from El Salvador (slightly greater length of teeth is due to a more full-length view). d: D. “gentica-like” from Jamaica. Note gap between teeth 3 and 4. e: Anterior (dorsal) view of D. gentica (LA), showing extensive dorsal membrane. Abbreviations: dscl, dorsal sclerite. Most drawings by D. Grimaldi. (TIF) [file pone.0122575.s002.tif]

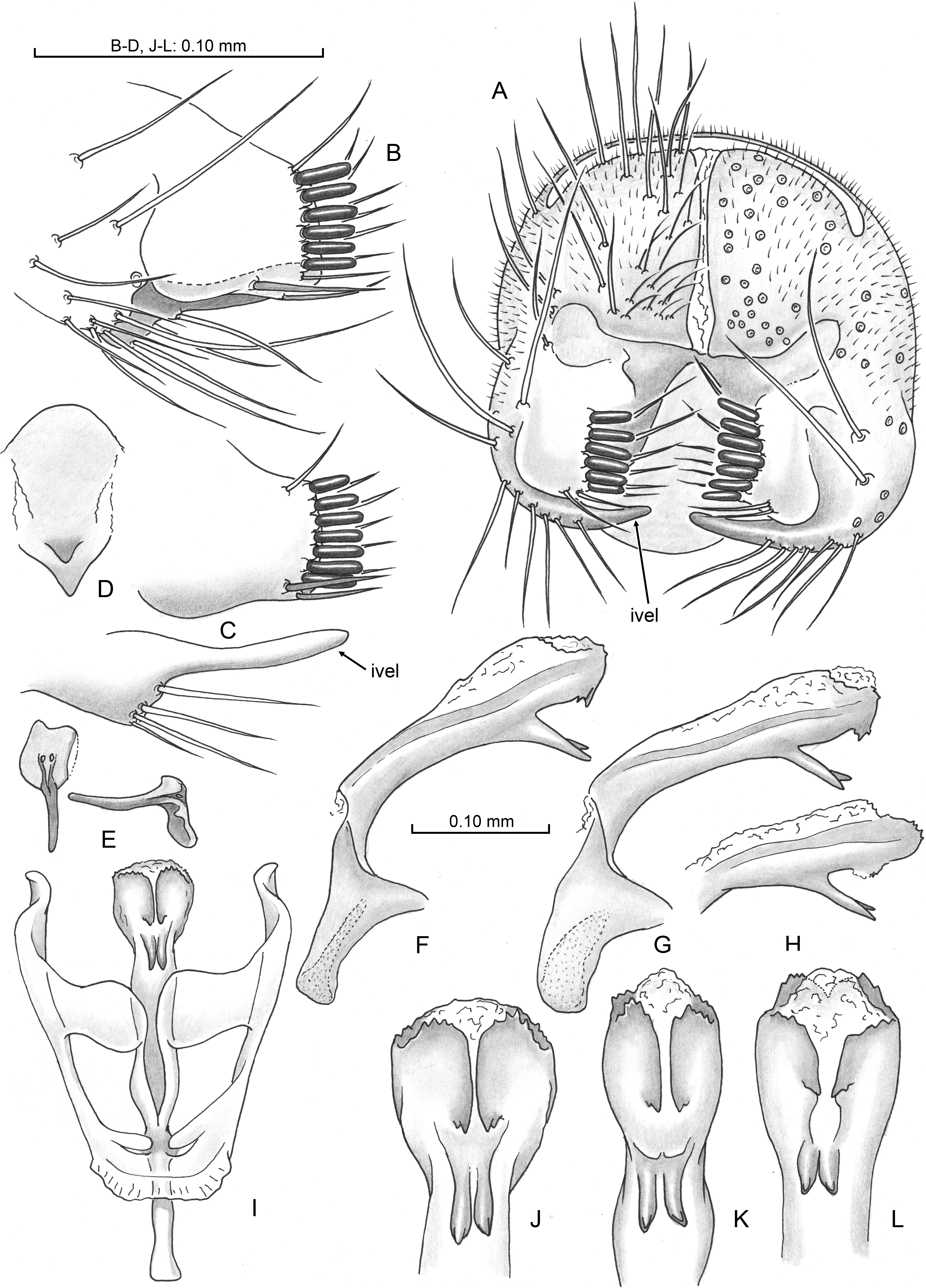

Supplement: S3 Fig — No male specimens of “gentica-like” from Jamaica were available. a: Epandrium and surstyli of LA specimen, posterior view. b, c: Detail of surstyli with ivel. b: LA specimen, with ivel intact and partially hidden. c: Paratype (El Salvador), with ivel disarticulated. d: Subepandrial sclerite of paratype (El Salvador). e: Ejaculatory apodeme in two views, paratype (El Salvador). f-h: Aedeagus plus aedeagal apodeme, lateral view. f: Paratype. g, h: LA specimens. i: Genitalia (aedeagus, hypandrium, aedeagal apodeme) of paratype (El Salvador), ventral view j-l: Apex of aedeagus (distiphallus), ventral view, of paratype (j) and LA specimens (k, l). Abbreviations: ivel, inner part of ventral epandrial lobe. Drawings by D. Grimaldi. (TIF) [file pone.0122575.s003.tif]

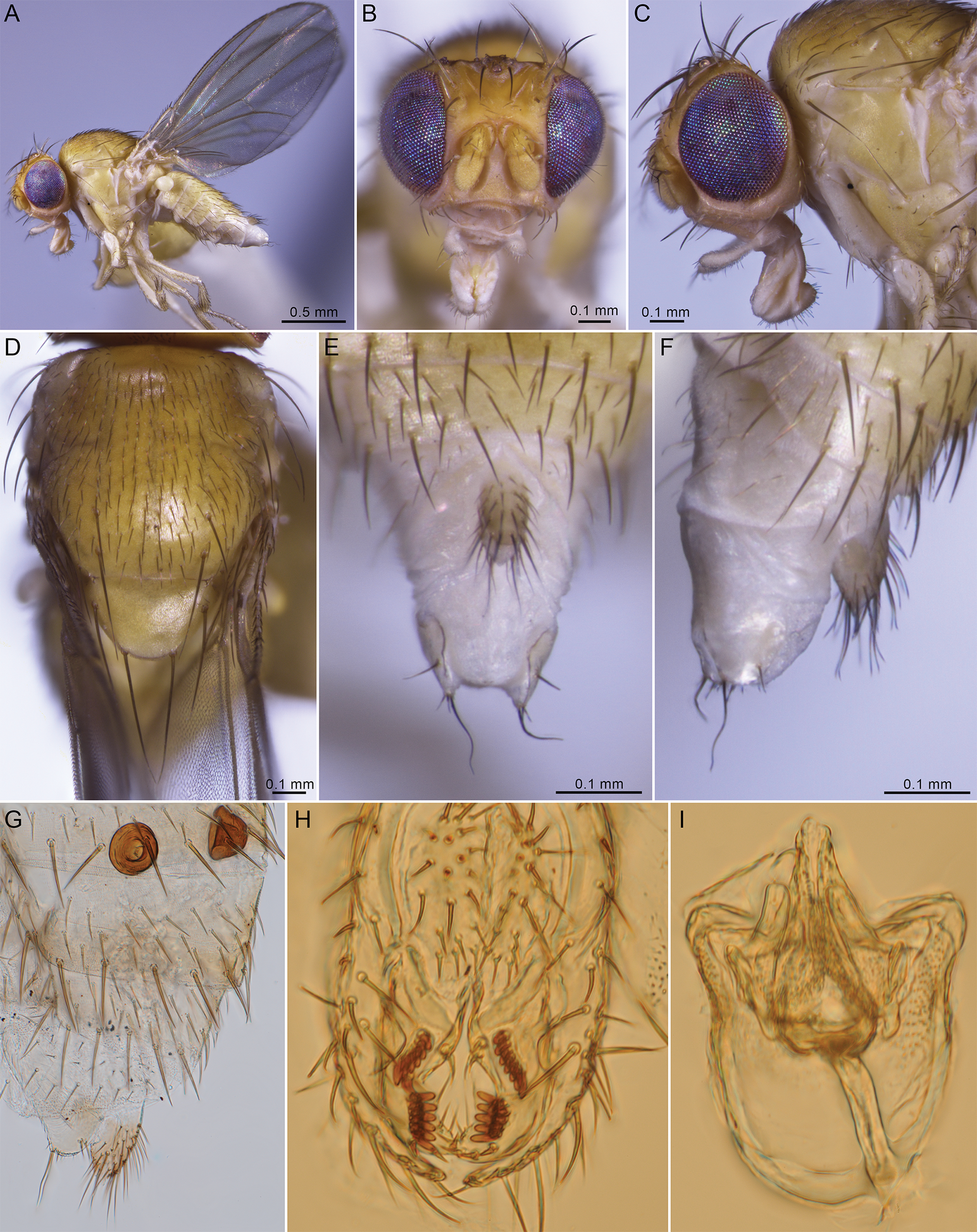

Supplement: S4 Fig — Specimens dried from ethanol using HMDS; all are from California. A. Lateral habitus. B. Frontal view of head. C. Lateral view of head and anterior portion of thorax. D. Thorax, dorsal view. E. Female terminalia, dorsal view. F. Ibid., lateral view. G. Female terminalia cleared, lateral view, showing spermathecae. H. Epandrium with surstyli. I. Hypandrium, aedeagus, periphallic structures. Photos by D. Grimaldi. (TIF) [file pone.0122575.s004.tif]

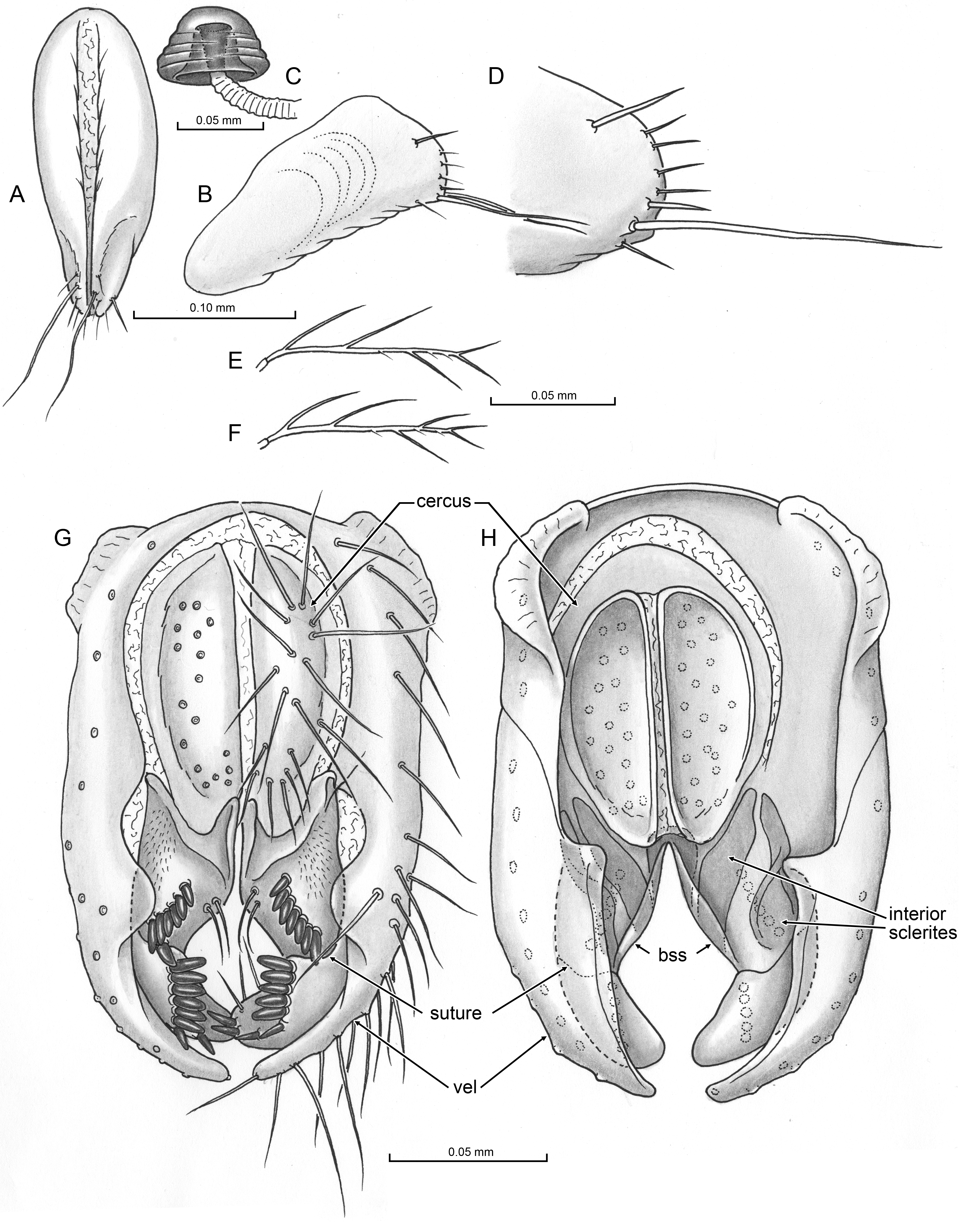

Supplement: S5 Fig — Specimens from Los Angeles. A-D: Female terminalia. A. Oviscapt, ventral view. B. Oviscapt, lateral view. C. Spermathecal capsule, lateral view. D. Oviscapt, detail of apex. E, F: Arista of two individuals. F. Arista of female, from sample 15608. G. Arista of male from sample 15438. H. Epandrium with cerci and surstyli, posterior view. I. Ibid., interior view (setae omitted). Abbreviations: bss, basal segment of surstylus; vel, ventral epandrial lobe. Drawings by D. Grimaldi. (TIF) [file pone.0122575.s005.tif]

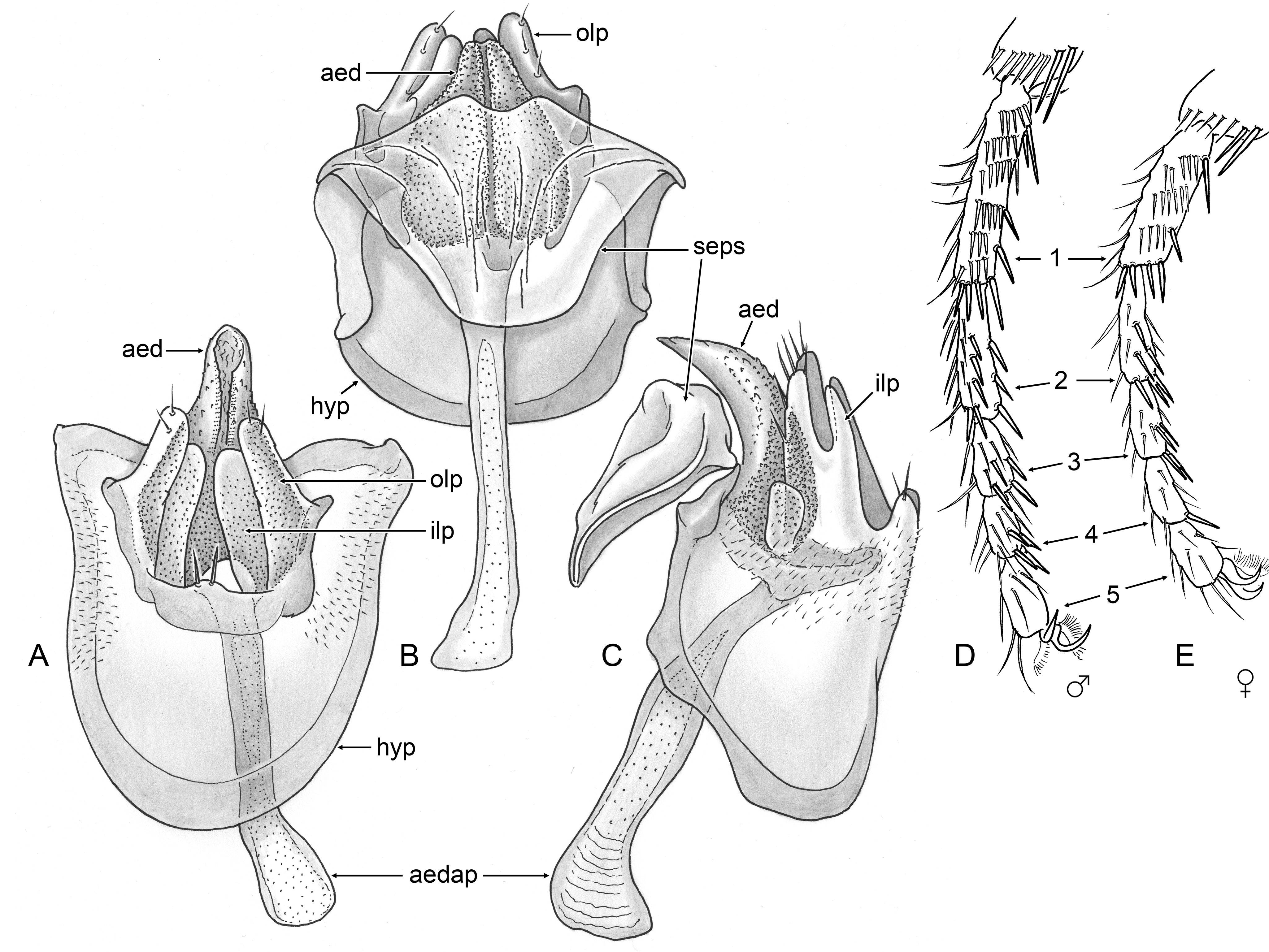

Supplement: S6 Fig — A-C: Drawings of internal male genitalia of D. flavohirta (LA specimens); D-E: male and female protarsi, oblique ventral views (Australian specimens). A. Hypandrium, aedeagus, paraphyses and aedeagal apodeme, ventral view. B. Ibid., dorsal view. C. Ibid., lateral view. D. Male protarsus of D. flavohirta. E. Female protarsus of D. flavohirta. Note pairs of slightly larger, but unsclerotized, setae on tarsomeres, and lack of sexual dimorphism in the setation. Abbreviations: 1–5: protarsomeres 1–5. aed, aedeagus; aedap, aedeagal apodeme; hyp, hypandrium; ilp, inner lobe of paraphysis; olp, outer lobe of paraphysis; seps, subepandrial sclerite. Photos and drawings by D. Grimaldi. (TIF) [file pone.0122575.s006.tif]

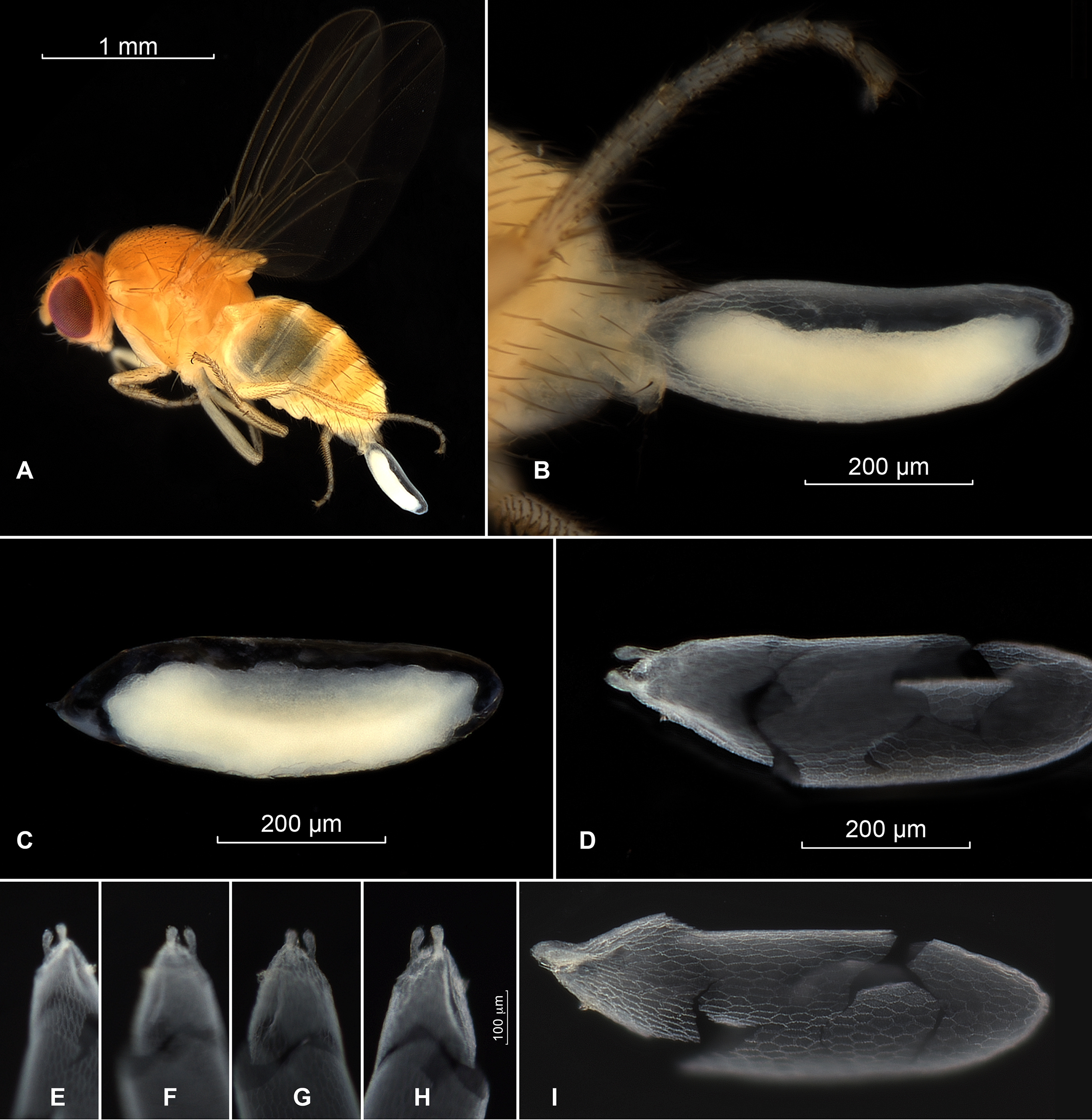

Supplement: S7 Fig — Specimens from Stroud, NSW, Australia. A, B: Female with partially extruded egg. The oviposition of single, large, mature eggs is a typical feature of anthophilous Drosophilidae (note well-developed embryo through the chorion in figs. B, C), as is the reduction of the anterior filaments of the egg. In this species, the pair of egg filaments typical of Sophophora are reduced to small, stubby, preapical lobes (Figs. D–I). Photos by S. F. McEvey. (TIF) [file pone.0122575.s007.tif]

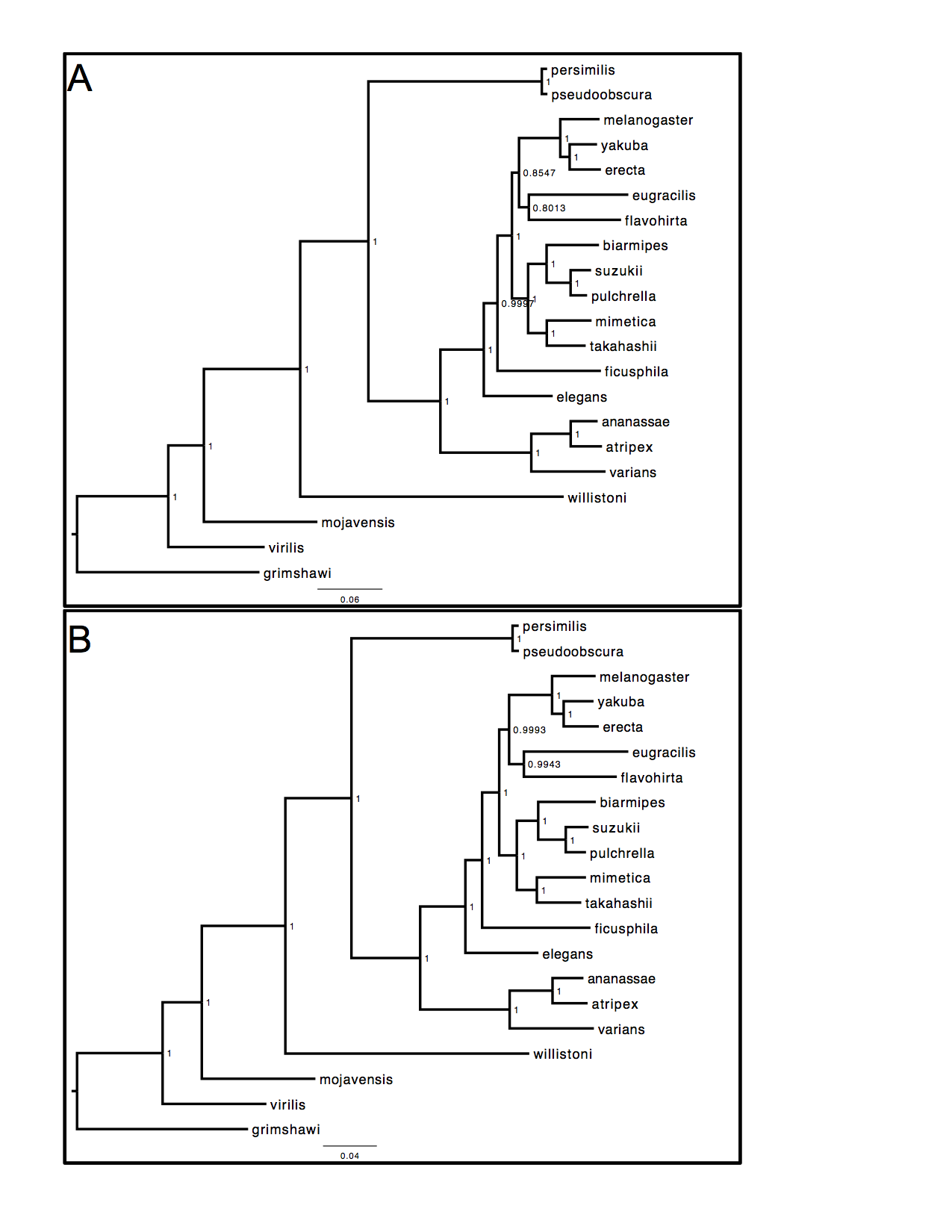

Supplement: S8 Fig — The phylograms focus on melanogaster group species and various outgroups. They are generated with (A) and without (B) third-position bases. Both analyses place D. flavohirta as sister to D. eugracilis with this pair sister to the melanogaster subgroup, relative to the other taxa considered. Numbers indicate posterior probabilities for node support. Phylograms by M. Turelli and P. Ginsburg. (TIF) [file pone.0122575.s008.tif]
