# Supplementary material for: Strange Little Flies in the Big City: Exotic Flower-Breeding Drosophilidae (Diptera) in Urban Los Angeles
Source: PLoS One. 2015 Apr 29;10(4):e0122575. doi: 10.1371/journal.pone.0122575 (PMC4414507; doi:10.1371/journal.pone.0122575)
Supplement: S1 Text — (DOCX) [file pone.0122575.s009.docx]

**Supporting Information**

**Systematics**

***Drosophila (Drosophila) gentica* Wheeler and Takada**

**S1-S3 Figs.**

*Drosophila gentica* Wheeler and Takada 1962, in [1]: 406. Type locality: San Salvador, El Salvador. Holotype male in U.S. National Museum of Natural History.

DESCRIPTION: General body coloration a light, dull yellowish to pale, infuscate brown, lighter ventrally. **Head**: Eye pale red to pink, with dense interfacetal setulae on lateral surface; ocellus light, area between ocelli light (not dark brown); frons very pale brownish, infuscate, frontal vitta dull, not shiny; frontal-orbital plate, face, antenna, palp, proboscis a lighter, dull yellow. All setae dark brownish-bronze (vs. black). Frontal index 1.03; frontal width index LA specimens 1.30 (1.23-1.38), *gentica* paratypes 1.45 (1.31-1.78). **Setation**: Ocellar setae divergent; postocellars convergent to parallel; inner (medial) verticals parallel to slightly convergent; outer (lateral) verticals strongly divergent, lateroclinate; large occipital seta immediately posterior to outer vertical, pair convergent. Orbital setae: Proclinates (or1) parallel to divergent; anterior reclinate (or2) relatively small, lying slightly closer to or1 than to post. reclinate (or3); or3 approximately midway between or1 and inner vertical seta, sometimes strongly lateroclinate; or2 lying slightly lateral to tangent between or1, or3 and inner vertical. Ratio or1-or3 0.81, or2-or1 0.50, postocellar index 0.51; ocellar index 0.75; vt-index 0.93. Face with single pair of vibrissae, with 5-6 much smaller setae on ventral margin of gena; cheek index 1.30. **Antenna**: Pedicel with 3 setae, numerous setulae; flagellomere 1 cream-colored to very light infuscate greyish-brown; arista with 3 dorsal and 2 ventral branches, plus minute terminal fork. **Face and proboscis**: Facial carina well developed, nose-like, edge rounded (not flat), without sulcus, thinner than basal flagellomere; facial color varying from homogeneous light to having ventral portion of carina and oral margin a pale brown. Palp light, cream-colored, with two setae on ventral margin. Proboscis with cream-colored labium; labellum narrow, slightly geniculate.

**Thorax**: Scutum and disk of scutellum yellow, slightly dull but not pollinose or shiny. Acrostichal setulae in regular rows, 6 rows between dorsocentral setae; acrostichal seta immediately anterior to ant. dorsocentral sometimes enlarged, to ca. 1.3X length and slightly thicker than other acrostichals, often unmodified. No prescutellar setae present; posterior dorsocentral larger than anterior dc, dc-index 1.82 (1.56-2.13) in LA specimens, in *gentica* paratypes 1.67 (1.50-2.00). Two or three large postpronotal setae present, h-index 1.11; anterior scutellars longer than post. scut, parallel, scutellar index 1.16, apical scutellars crossing for ca. 0.6-0.7X their length. Three notopleural setae present, dorsal one longest; 3 supra-alar setae, posterior one >2X length of others. Pleuron lighter than scutum; bare except for katepisternum, with several setulae and one long posterior seta, shorter anterior one, sterno-index (ant./post. katepisternal setae) 0.50. **Legs**: Very light, cream-colored, slightly lighter than pleuron. Fore femur with 2-3 longer lateral setae, 2-3 longer ventral setae; fine setulae on all leg segments in regular longitudinal rows, setulae shorter, denser on tarsomeres. Strong ventral-apical seta on mid tibia, finer one dorsally; fine, erect, preapical seta dorsally on hind tibia. Fore tarsi of males without fine, erect setulae on dorsal surface. **Wing**: Completely hyaline (no markings), fairly slender (wing index [length/width] 2.20), tip faintly pointed; C-index 0.23; ac-index in LA specimens 4.17 (3.90-4.41), in *gentica* paratypes 3.71 (3.47-3.87); 4C-index 2.86; 4V- index 2.05; 5X- index in LA specimens 5.75 (5.52-6.00), *gentica* paratypes 6.52 (5.44-7.69); M-index 2.31.

**Abdomen**: Very light, dusky, yellowish-brown, with no distinct bands, spots or other markings; tergites with setae fairly short and sparse, ones on posterior margins slightly longer. **♂ Terminalia**: Epandrium same color as tergites, dorsally with microtrichia and scattered setae; cercus yellow, laterally connected to epandrium, with abundant large setae and ca. 10 finer, small ones on ventral margin; ventromedial corner of cercus slightly pointed. Ventral lobe of epandrium bare of microtrichia, with ca. 12 long setae (2 dorsal ones longest); lobe elongate, bent inward (ventromedially), apical half sclerotized and pointed. Surstylus broad, connected to ventral lobe of epandrium, bare of microtrichia; inner margin with row of 6 long, sclerotized prensisetae (lengths ca. 3X the width), tips rounded, sometimes with thin, sharp 7^th^ (ventralmost) prensiseta; two thick setae ventrally. Subepandrial sclerite well developed, approximately oval, with pointed anterior margin. Hypandrium in full ventral view trapezoidal, faintly sclerotized gonopods present, with minute pair of apical setae medially. Aedeagus relatively short, ca. 200 µm in length, slightly curved in lateral view; apex bulbous (more so in lateral view), with pair of preapical ventral spines pointed apicad; tip of aedeagus with fine serrations ventrally and dorsally; dorsum of distiphallus membranous. Aedeagal apodeme ca. 0.4X length of aedeagus, keel-like; ejaculatory apodeme present, structure like a mason palette.

**♀ Terminalia**: Dorsal surface of penultimate tergite (tVII) incomplete (membranous) medially. Apical tergite (tVIII) with microtrichia only on dorsal surface; lateral surfaces glabrous, curved downward, with small partially-articulating sclerite on each side; epi-/hypoprocts setose, area between these and oviscapt extensively membranous. Oviscapt short, broad in lateral view, stout; with row of 6 long, pointed, heavily sclerotized teeth on inner-apical margin of each valve; each oviscapt tooth on slightly raised base, but bases not pedunculate; teeth evenly spaced, interdigitate on opposing valves. Dorsal membranous surface of oviscapt with dense, minute microtrichia/spicules and with pair of sclerites, roughly triangular in shape. Spermatheca relatively small (ca. 100 µm diameter), heavily sclerotized, spherical; lacking introvert; with equatorial band of short striations, apical half with faint, minute papillae; spermathecal duct very narrow.

**S1 Fig.** **Photomicrographs of *Drosophila gentica* Wheeler and Takada collected in Los Angeles (Figs. 1a-f, h-j), and of *Drosophila* “near *gentica*” from Jamaica (Fig. 1g: see text)**. a, b: Female, lateral views. c, d: male, dorsal views of thorax (c) and abdomen (d). Unlabelled arrows in c point to enlarged acrostichals in front of *adc* setae. e-g: frontal views of head. e, f: Male *D. gentica* from LA (note light brown oral band on specimen in f). g: Female specimen from Bath, Jamaica (note broader, shorter carina). h-j: Terminalia of flies from LA. h: Male, lateral view, with aedeagus everted. i: Female, lateral view. j: Female, terminal view. Abbreviations: *adc,* anterior dorsocentral seta; *pdc*, posterior dorsocentral seta. Photos by D. Grimaldi

**S2 Fig.** **Terminalia of *Drosophila gentica* and *gentica*-like species.** A-E: Female terminalia of *Drosophila gentica* and near-*gentica* from Jamaica. F, G: Original drawings of male genitalia from Wheeler et al. (1962: their figures II-10 and III-9). f: “*gentica*-like” from Jamaica. g: *D. gentica* paratype, El Salvador. A-E: Drawings of female terminalia of *Drosophila gentica* specimens from El Salvador (c) and Los Angeles (a, b, e), and “*gentica*-like” species from Jamaica (d). a: Spermatheca of *Drosophila gentica*. b-d: Posterior (terminal) views of oviscapt. b: Terminal abdominal sclerites of *D. gentica* from Los Angeles. c: Teeth on left valve of *D. gentica* paratype from El Salvador (slightly greater length of teeth is due to a more full-length view). d: *D*. “*gentica*-like” from Jamaica. Note gap between teeth 3 and 4. e: Anterior (dorsal) view of *D. gentica* (LA), showing extensive dorsal membrane. Abbreviations: dscl, dorsal sclerite. Most drawings by D. Grimaldi.

**S3 Fig.** **Drawings of male terminalia of *Drosophila gentica* specimens from El Salvador (paratype series) (c, d, e, f, i, j) and newly collected from Los Angeles (a, b, g, h, k, l).** No male specimens of “*gentica*-like” from Jamaica were available. a: Epandrium and surstyli of LA specimen, posterior view. b, c: Detail of surstyli with *ivel*. b: LA specimen, with *ivel* intact and partially hidden. c: Paratype (El Salvador), with *ivel* disarticulated. d: Subepandrial sclerite of paratype (El Salvador). e: Ejaculatory apodeme in two views, paratype (El Salvador). f-h: Aedeagus plus aedeagal apodeme, lateral view. f: Paratype. g, h: LA specimens. i: Genitalia (aedeagus, hypandrium, aedeagal apodeme) of paratype (El Salvador), ventral view j-l: Apex of aedeagus (distiphallus), ventral view, of paratype (j) and LA specimens (k, l). Abbreviations: *ivel*, inner part of ventral epandrial lobe. Drawings by D. Grimaldi.

***Drosophila (Sophophora) flavohirta* Malloch**

**S4-S7 Figs.**

*Drosophila flavohirta* Malloch, 1924 [2]: 354. Type locality: Como, New South Wales, Australia. Holotype female in Australian Museum, Sydney (AM K73328).

**DESCRIPTION: HEAD**: Broader than deep; entirely light yellowish (except for eyes). Frontal index 0.94, frontal width index 1.44. Eye with short, dense micropubescence; eye varying in color depending on preservation (pink in air-dried specimens; iridescent greenish in live and in ethanol-preserved specimens; iridiscent purplish-green in HMDS solvent-dried specimens), fading from natural color days after drying; ocelli light; eye depth/width 1.13. Frontal vitta dull, microscopically striate; ocellar triangle and frontal-orbital plate slightly pollinose. Cheek relatively shallow. **Setation**: Postocellars parallel to convergent (but not crossing), postocellar index 0.49; ocellars parallel, base of ocellar slightly outside of tangent between median and lateral ocellus, ocellar index 0.69. Inner vertical strongly inclinate; outer vertical lateroclinate, outer vertical slightly longer; one pair of inclinate postverticals, length ca. 0.5X that of outer vertical. Proclinate orbital with base directly medial to anterior reclinate; bases separated by distance approximately twice diameter of setal sockets. Anterior reclinate slightly shorter than other orbitals, orbital seta 1-3 ratio 0.92, 2-1 ratio 0.75; posterior reclinate orbital slightly closer to anterior reclinate than to inner vertical seta. Few, small setulae on anterior portion of frontal-orbital plates and front. One large pair vibrissae present; vibrissa light, almost white; subvibrissa ca. 0.5X length of vibrissa; cheek with row of four fine setae posterior to vibrissa. **Antenna**: Pedicel setulose on antero-dorsal surface; one strong seta near middle, two near margin. Flagellomere I short, broadly oval; with fine, short setulae; flagellomere II yellow, minute, ring-like segment at base of arista; arista short plumose, with 2-3 short dorsal branches (apical-most one absent in some individuals) and one ventral branch (plus very short apical fork); ventral branch between 2^nd^ and 3^rd^ dorsal branches. Length of longest aristal branch ca. 0.5X the length of main arista branch. **Face and proboscis.** Face raised but not carinate; raised between antennae and flaring out to raised portion of oral margin; antenna in a recessed area; face lighter than front. Proboscis short, largely concealed inside oral cavity when retracted, labellum small and setose; palps very light, length approximately equal to that of theca, with 1 apical and 2-3 very fine apical/subapical setae.

**THORAX**: Length 0.78 mm. Scutum and scutellum light yellow, pleura and coxae lighter, nearly white. Acrostichals in 6-8 rows; ones near scutellar margin very slightly enlarged to not enlarged at all. Postpronotal lobe with two long setae, ratio upper/lower seta lengths (H-index) 0.81. Two pairs well-developed dorsocentrals, anterior dc significantly smaller: ratio ant./post. dc seta lengths 0.57; ratio dc seta distances 2.40. Three notopleural setae present, two larger ones on notopleural margin. Scutellar setae long, posterior pair slightly convergent to having tips cruciate; scutellar index 0.85. Two katepisternal setae present, posteroventral one slightly longer, sterno-index 0.77. Two supra-alar setae present, posterior one longer. **Legs**: Very light yellow, no coloration patterns. Profemur with 6-7 partially erect setae on distal third (3-4 ventrally, 2 laterally, 1 dorsal); protibia with 1 short ventral and 1 short dorsal preapical erect seta. Very little sexual dimorphism in protarsi, principally in length of probasitarsomere: female probasitarsomere 0.32X total length of protarsus, male 0.37X. Male without large, black, sclerotized sexual combs on protarsus. Both sexes with thickened setae on protarsomeres, but no detectable differences in number, positions, or thickness of these setae between sexes. Mesofemur with 3-4 longer preapical setae; mesotibia with 1 short, erect dorsal preapical seta, 1 long ventroapical seta (length slightly greater than greatest width of tibia); immediately proximal to base of dorsal preapical seta on mesotibia is transverse comb of 5-6 short, stout, black setae (setae thicker in male). Metafemur without distinctive setae; metatibia with 1 fine, short, erect preapical seta on dorsal surface. Tarsomeres on all leg pairs with bare ventral surface, bordered by longitudinal rows setae; each tarsomere with pair of short, stout, dark setae on ventroapical margin. **Wing**: Length 1.61 mm; ratio length/width 2.26; completely hyaline; veins light yellow to transparent; costal spinules black, densely-packed, end slightly past mid-way between apices of veins R_2+3_ and R_4+5_. Veins R_4+5_ and M_1+2_ parallel, crossvein dm-cu significantly oblique (not perpendicular) to M; anal vein present, anal lobe well developed. C-index 0.37; ac-index 2.05; 4C-index 2.80; 4V-index 2.73; 5X-index 7.87; M-index 2.77.

**ABDOMEN**: Tergites light yellow, but with faint greenish transverse band on each that fades quickly after death; sternites lighter (nearly white); tergites setose, with row of longer setae on posterior margin of each. **Male Terminalia**: Epandrium setose but without microtrichia; with pair of short phragmas on dorsolateral corners, lateral walls wrapped anteriad, particularly walls of ventral lobe; with pair of well-formed condyles anteriorly, which articulate with posterolateral corners of hypandrium and/or subepandrial sclerite. Epandrium depth 1.6X the width; ventral lobe of epandrium long, pendulous, length 0.5X depth of epandrium, lobe setose, with shallow, rounded lobe on mesal surface. Cercus not fused laterally to epandrium, devoid of microtrichia, with membranous rim, pair of shallow ventral lobes. Surstylus large, pendulous, base fused to epandrium beneath cercus; surstylus 2-segmented, with fine suture between two groups of dark, heavily sclerotized prensisetae; dorsal group with row of 7 prensisetae, diagonal to ventral row of 5-6 prensisetae; 4 smaller prensisetae (2 apical, 2 subapical), plus some fine setae. Surstylus partially nestled within concave ventral lobe of epandrium. Subepandrial sclerite large, faintly sclerotized, roughly pentagonal in shape; sclerite covers much of aedeagus and paraphyses; anterior portion slightly emarginate; lateral margins produced into pair of thin condyles, articulate with condyles on epandrium. Hypandrium U-shaped, ca. 1.5X length of aedeagus, with patch of microtrichia laterally; paramedian pair of fine, minute setulae on posterior margin. Aedeagus roughly conical in shape, short, bilobed, with scattered fine scales apically and minute papillae overall. Paraphysis (postgonite) bilobed, both lobes digitiform, inner lobe slightly shorter; outer lobe with row of 3 fine setulae on distal half. Aedeagal apodeme long, slender, straight; length ca. 1.3X that of aedeagus. Ejaculatory apodeme either extremely vestigial or entirely lost. **Female Terminalia**: Tergite VII very light, barely sclerotized, divided dorsomedially. Oviscapt well developed, but very lightly sclerotized and without pegs, with fine setae and setulae only (apically and subapically); in ventral view oviscapt narrowly oval, laterally broad with blunt apex bearing ca. 5 minute, fine setulae and long, fine seta that is 0.5X length of oviscapt; oviprovector membrane without scales. Spermathecal capsule heavily sclerotized, cap-shaped, height 0.6X the width, with fine annulations; length of introvert 0.7X the height of capsule. **Egg**: Large, length ca. 500 μm, ca. 0.25X length of fly; with well developed, mature embryo at time of oviposition; anterior end with pair of short, stubby, preapical lobe-like egg filaments, length ca. 50 μm.

**S4 Fig**. **Photomicrographs of *Drosophila (Sophophora) flavohirta* Malloch. S**pecimens dried from ethanol using HMDS; all are from California. A. Lateral habitus. B. Frontal view of head. C. Lateral view of head and anterior portion of thorax. D. Thorax, dorsal view. E. Female terminalia, dorsal view. F. Ibid., lateral view. G. Female terminalia cleared, lateral view, showing spermathecae. H. Epandrium with surstyli. I. Hypandrium, aedeagus, periphallic structures. Photos by D. Grimaldi.

**S5 Fig**. **Drawings of terminalia and other characters of *D. flavohirta*.** Specimens from Los Angeles. A-D: Female terminalia. A. Oviscapt, ventral view. B. Oviscapt, lateral view. C. Spermathecal capsule, lateral view. D. Oviscapt, detail of apex. E, F: Arista of two individuals. F. Arista of female, from sample 15608. G. Arista of male from sample 15438. H. Epandrium with cerci and surstyli, posterior view. I. Ibid., interior view (setae omitted). Abbreviations: *bss*, basal segment of surstylus; *vel*, ventral epandrial lobe. Drawings by D. Grimaldi.

**S6 Fig**. **Structures of *Drosophila flavohirta***. A-C: Drawings of internal male genitalia of *D. flavohirta* (LA specimens); D-E: male and female protarsi, oblique ventral views (Australian specimens). A. Hypandrium, aedeagus, paraphyses and aedeagal apodeme, ventral view. B. Ibid., dorsal view. C. Ibid., lateral view. D. Male protarsus of *D. flavohirta*. E. Female protarsus of *D. flavohirta*. Note pairs of slightly larger, but unsclerotized, setae on tarsomeres, and lack of sexual dimorphism in the setation. Abbreviations: *1-5*: protarsomeres 1-5. *aed,* aedeagus; *aedap*, aedeagal apodeme; *hyp*, hypandrium; *ilp*, inner lobe of paraphysis; *olp*, outer lobe of paraphysis; *seps*, subepandrial sclerite. Photos and drawings by D. Grimaldi.

**S7 Fig.** **The unusual eggs of *Drosophila flavohirta***. Specimens from Stroud, NSW, Australia. A, B: Female with partially extruded egg. The oviposition of single, large, mature eggs is a typical feature of anthophilous Drosophilidae (note well-developed embryo through the chorion in figs. B, C), as is the reduction of the anterior filaments of the egg. In this species, the pair of egg filaments typical of *Sophophora* are reduced to small, stubby, preapical lobes (Figs. D-I). Photos by S. F. McEvey.

**Molecular Phylogenetic Analyses including *D. flavohirta***

**S8 Fig.**

Phylogenetic analyses combining old and new sequence data were conducted to increase resolution of the placement of *D. flavohirta* in relation to the *melanogaster* subgroup within the *melanogaster* species group. Partial coding sequences were obtained for 12 nuclear and 2 mitochondrial loci (nuclear: *Alcohol dehydrogenase* (Adh), *Amylase* (Amy), *Amylase-related* (Amyrel), *Dopa decarboxylase* (Ddc), *extra sexcombs* (esc), *Glycerol 3 phosphate dehydrogenase* (Gpdh), Histone 2 spacer (H2s), *hunchback* (hb), *kinase suppressor of ras* (ksr), *Phospho-glucose isomerase* (Pgi), *Triose phosphate isomerase* (Tpi), and *Xanthine dehydrogenase* (Xdh); mitochondrial: cytochrome oxidase subunit 1 (CO1) and cytochrome oxidase subunit 2 (CO2)), making a total of 9 to 14 loci per species, sequenced across 21 species. The concatenated sequence (when including all fourteen loci) totaled to 9757 bp. Taxa were chosen whose relationships to *D.* *flavohirta* conflicted among previous studies or showed low statistical support (as discussed in the main text). Specifically, representative taxa were chosen from the analysis by [3] within the *melanogaster* subgroup and the “oriental” lineage (e.g., the *suzukii, elegans* subgroups, etc.) as well as various outgroups.

**S8 Fig**. **Bayesian phylograms including *Drosophila flavohirta***. The phylograms focus on *melanogaster* group species and various outgroups. They are generated with (A) and without (B) third-position bases. Both analyses place *D. flavohirta* as sister to *D. eugracilis* with this pair sister to the *melanogaster* subgroup, relative to the other taxa considered*.* Numbers indicate posterior probabilities for node support. Phylograms by M. Turelli and P. Ginsburg.

Bayesian analyses were performed using MrBayes 3.2 [4]. For all analyses, the concatenated sequence was analyzed using the GTR +Γ substitution model, partitioned by gene and unlinked to allow independent parameter estimation for each gene region. Each MCMC was run with four chains for 2500000 generations. MCMC performance was assessed visually for adequate convergence of parameter estimates using time-series plots in Tracer v1.6 [5]. Tree convergence among runs was assessed with the average standard deviation of split frequencies sampled every 5000 generations, using a threshold value for adequate convergence between runs of ≤ 0.1.

The phylograms resulting from two analyses are shown in S8 Fig., both provide strong support for a clade ((*D. flavohirta*, *D. eugracilis*), (the *melanogaster* subgroup)) that excluded other “oriental” lineage species considered. The first analysis included all of the data, and resulted in a posterior probability of 0.8547 for the clade ((*D. melanogaster* (*D. yakuba, D. erecta*), (*D. flavohirta, D. eugracilis*)) and a posterior probability of 0.8013 for the sister relationship between *D. flavohirta* and *D. eugracilis*. S8A Fig. shows relatively long branches associated with *D. flavohirta* and *D. eugracilis*, and relatively short branches for outgroup species (e.g., *D. grimshawi*). We conjectured that long-branch attraction artifacts [6, 7] associated with saturation of differences at third-codon positions might contribute to the relatively weak resolution of the relationships of *D. flavohirta* and *D. eugracilis* to one another and the *melanogaster* subgroup. After excluding data from the third codon position, the revised analysis (S8B Fig.) gave posterior probabilities of 0.9943 for the clade (*D. flavohirta, D. eugracilis*) and 0.9993 for the clade ((*D. melanogaster* (*D. yakuba, D. erecta*)), (*D. flavohirta, D. eugracilis*)). These results strengthen previous phylogenetic analyses indicating that *D. flavohirta* is closely related to the *melanogaster* subgroup. They also suggest a novel resolution of the two uncertain nodes involving *D. flavohirta*  and *D. eugracilis* in Supplemental Figure 1 of [3], placing them as sisters relative to the other taxa included.

To our knowledge, *D. flavohirta*’s proposed relationship to *D. eugracilis* has not been previously suggested. Notably, *D. eugracilis* and *D. flavohirta* possesses similar male genitalia [8]. Both have a two-segmented surstylus, each with rows of prensisetae separated by a suture. In *D. flavohirta*, the prensisetae are in rows of 7 prensisetae dorsally and 5-6 ventrally. In contrast, *D. eugracilis* possess prensisetae in rows of 9 dorsally and 5 ventrally. In addition, *D. eugracilis* possess a greatly reduced sex comb on the male protarsus with respect to other *melanogaster* group species, consisting of only two enlarged bristles. Further examination of *D. eugracilis* and *D. flavohirta* may reveal additional potential synapomorphies.

**Supporting Information References**

**1.** Wheeler MR, Takada H, Brncic D. XIV. The *flavopilosa* species group of *Drosophila*. Univ Texas Publ Genetics 1962; 6205: 395-414.

**2.** Malloch JR. Notes on Australian Diptera. No. IV. Proc Linn Soc NSW 1924; 49: 348-359.

**3.** Barmina O, Kopp A. Sex-specific expression of a HOX gene associated with rapid morphological evolution. Developmental Biol 2007; 311: 277-286.

**4.** Ronquist F, Huelsenbeck JP. MRBAYES 3: Bayesian phylogenetic inference under mixed models. Bioinformatics 2003; 19:1572-1574.

**5.** Rambaut A, Suchard MA, Xie D, Drummond AJ. Tracer v1.6, 2014; Available from <http://beast.bio.ed.ac.uk/Tracer>

**6.** Felsenstein J. Cases in which parsimony or compatibility methods will be positively misleading. Systematic Zoology 1978; 27:401-410.

**7.** Bergsten J. A review of long-branch attraction. Cladistics 2005; 21:163-193.

**8.** Bock I, Wheeler MR. The *Drosophila* *melanogaster* species group. Austin, Texas: University of Texas Publication 1972; 7213:1-102.
